# Unraveling the key to the resistance of canids to prion diseases

**DOI:** 10.1371/journal.ppat.1006716

**Published:** 2017-11-13

**Authors:** Natalia Fernández-Borges, Beatriz Parra, Enric Vidal, Hasier Eraña, Manuel A. Sánchez-Martín, Jorge de Castro, Saioa R. Elezgarai, Martí Pumarola, Tomás Mayoral, Joaquín Castilla

**Affiliations:** 1 CIC bioGUNE, Parque tecnológico de Bizkaia, Derio, Bizkaia, Spain; 2 Laboratorio Central de Veterinaria (LCV), Madrid, Spain; 3 Centre de Recerca en Sanitat Animal (CReSA), UAB-IRTA, Campus de la Universitat Autònoma de Barcelona, Bellaterra, Barcelona, Spain; 4 Servicio de Transgénesis, Nucleus, Universidad de Salamanca, Salamanca, Spain; 5 IBSAL, Instituto de Investigación Biomédica de Salamanca, Salamanca, Spain; 6 Department of Infectology, Scripps Florida, Jupiter, Florida, United States of America; 7 Department of Animal Medicine and Surgery, Veterinary faculty, Universitat Autònoma de Barcelona (UAB), Bellaterra (Cerdanyola del Vallès), Barcelona, Spain; 8 Centro de Investigación Biomédica en Red en Bioingeniería, Biomateriales y Nanomedicina (CIBER-BBN), Madrid, Spain; 9 IKERBASQUE, Basque Foundation for Science, Bilbao, Bizkaia, Spain; Creighton University, UNITED STATES

## Abstract

One of the characteristics of prions is their ability to infect some species but not others and prion resistant species have been of special interest because of their potential in deciphering the determinants for susceptibility. Previously, we developed different *in vitro* and *in vivo* models to assess the susceptibility of species that were erroneously considered resistant to prion infection, such as members of the *Leporidae* and *Equidae* families. Here we undertake *in vitro* and *in vivo* approaches to understand the unresolved low prion susceptibility of canids. Studies based on the amino acid sequence of the canine prion protein (PrP), together with a structural analysis *in silico*, identified unique key amino acids whose characteristics could orchestrate its high resistance to prion disease. Cell- and brain-based PMCA studies were performed highlighting the relevance of the D163 amino acid in proneness to protein misfolding. This was also investigated by the generation of a novel transgenic mouse model carrying this substitution and these mice showed complete resistance to disease despite intracerebral challenge with three different mouse prion strains (RML, 22L and 301C) known to cause disease in wild-type mice. These findings suggest that dog D163 amino acid is primarily, if not totally, responsible for the prion resistance of canids.

## Author summary

Detection of individuals or whole species resistant to any infectious disease is vital to understand the determinants of susceptibility and to develop appropriate therapeutic and preventative strategies. Canids have long been considered resistant to prion infection given the absence of clinical disease despite exposure to the causal agent. Through extensive analysis of the canine prion protein we have detected a key amino acid that might be responsible for their universal resistance to prion disease. Using *in vitro* and *in vivo* models we demonstrated that the presence of this residue confers resistance to prion infection when introduced to susceptible animals, opening the way to develop a new therapeutic approach against these, at present, untreatable disorders.

## Introduction

Prion diseases or transmissible spongiform encephalopathies (TSEs), a group of fatal neurodegenerative disorders, have been described since the XVIII^th^ century when clinical signs of scrapie in sheep were reported in England [1]. Prion disorders are caused by misfolding of a protein, the cellular prion protein (PrP^C^), which is expressed abundantly in the central nervous system, into an aggregated, self-propagating, disease-associated isoform known as PrP^Sc^ [2, 3]. Prion diseases occur worldwide and affect many different mammalian species including humans. Natural prion disorders include scrapie in sheep and goats, bovine spongiform encephalopathy (BSE) in cattle, transmissible mink encephalopathy (TME) in mink, chronic wasting disease (CWD) in cervids, and Kuru, Creutzfeldt-Jakob disease (CJD), Gerstmann-Straussler-Scheinker syndrome (GSS), fatal familial insomnia (FFI) and variably protease-sensitive prionopathy (VPSPr) in humans, which arise either sporadically–putatively spontaneous misfolding of PrP^C^–or are caused by mutations in the PrP encoding gene that are inherited as an autosomal dominant trait [4, 5]. Additionally, as a consequence of the BSE epidemic which occurred in the United Kingdom during the 1990’s, several mammalian species became infected with BSE prions. Such natural interspecies prion transmission, which had never been reported previously, gave rise to several new prion diseases including feline spongiform encephalopathy (FSE) in several feline species, TSE in a small number of non-human primates (NHP) and exotic ungulate spongiform encephalopathy (EUE) in several species of exotic ruminants of the *Bovidae* family kept in captivity [6, 7]. These disorders appeared as a consequence of the consumption of BSE, originating from cattle, contaminated food in a way similar to that of variant CJD (vCJD) in humans [8]. The number of species known to be susceptible to TSE increased also due to experimental infections performed during scientific investigations which demonstrated that many rodent species, such as mouse, rat, hamster, guinea pig and bank vole can be infected experimentally by prions, as well as several non-human primates, e.g. chimpanzees, marmoset monkeys, stump-tail macaques, gibbons, spider monkeys, sooty mangabey, pigtail, cynomolgus and rhesus macaques, and squirrel monkeys [4, 9, 10]. Pigs, also of interest, due to their widespread consumption by humans, have also been shown to be susceptible to infection by experimental challenge [11, 12]. Even rabbits, a species thought resistant to prion diseases, have been infected experimentally with TSE [13]. Thus, a wide variety of animal species are susceptible to prion disease including members of several mammalian families such as *Bovidae*, *Cervidae*, *Muridae*, *Mustelidae*, *Felidae*, *Cricetidae*, *Caviidae*, *Leporidae*, *Suidae* and *Hominidae* along with other primate families.

PrP amino acid sequences are highly conserved among mammals with approximately 85% sequence, or greater, identity with human PrP except for marsupials, such as the opossum or wallaby, which have approximately 70% sequence identity. Reptile, fish and avian PrPs have sequence identities ranging from 18% to 35% compared to humans. Among the animal species susceptible to TSEs, the most similar sequence identity to human PrP is the chimpanzee with a 99.2% and the most distant is the cat with an 84.7% similarity [14–19]. However, the susceptibility of each species to TSEs has no correlation with PrP sequence divergence or its misfolding proneness as it is determined structurally [20]. TSE susceptibility predictions are complicated further as the differences in species related PrP^C^ misfolding capacity depend on the disease-associated prion strain to which they are exposed. Natural selection may have succeeded in making some individuals or a whole species highly or completely resistant to some diseases. Although there is evidence to suggest that complete resistance to TSEs may be a utopia [21], the susceptibility of some species is so low—due to a PrP sequence that is highly resistant to misfolding–that they become of great scientific interest. This is the case for rabbits [22], horses [23] and dogs [24] which were considered prion resistant species based on the lack of natural cases reported and negative results in those challenged experimentally with prions [25]. However, the susceptibility of every mammalian species has not been evaluated and therefore, theoretically, the existence of resistant species sympatric with susceptible ones remains a possibility such as species not present in UK zoos during the BSE epidemic or that were never challenged [e.g. pronghorns (*Antilocapra americana*)]. The absence of naturally occurring TSE cases or unsuccessful experimental challenges is not enough to classify any mammalian species as resistant to prion diseases as shown when rabbits and transgenic mice expressing rabbit PrP were proven definitively susceptible to TSE [13, 26]. Also, equine PrP has been shown capable of misfolding into a pathogenic isoform but only transiently or unable to be transmitted between individuals [27].

Absolute resistance of a species to TSEs should not be considered as such, rather, different levels of susceptibility should be considered, and these can be used to study the molecular mechanisms behind the low susceptibility of certain species to prion disorders. Given the idiosyncrasies of the causal agent of TSEs, the mechanism of the low susceptibility in some species may be due to a specific feature(s) of each PrP sequence and its misfolding proneness [28]. Comparative alignments of PrP sequences—taking into account the disease susceptibility of each species—is one of the preferred methods to search for low susceptibility determinants [19, 29–33].

Leaving aside rabbits and horses as their PrPs can be converted in to pathogenic isoforms [13, 27], members of the *Canidae* family and in particular domestic dogs (*Canis lupus familiaris*), are the most interesting species to study as their PrP has never been shown to misfold *in vivo* [34]. Despite some studies or reports related to the susceptibility of dogs to prion disorders [35–37], most of them were never confirmed, were simple reports with no scientific procedure involved or were purely speculative.

All the members of the *Canidae* family, which includes wolves, foxes, jackals, dingoes and domestic dogs, share an almost identical PrP sequence with few polymorphic variants. Among the polymorphisms, the presence of either Aspartic acid or Glutamic acid in position 163 draws specific attention as it is highly characteristic and almost unique to the *Canidae* family [19]. Moreover, when compared to susceptible species or to those of unknown susceptibility, dog PrP presents several characteristics of interest as possible determinants for its low misfolding proneness [38, 39].

Thus, domestic dog PrP was chosen to study the possible causes of apparent resistance to misfolding of PrP from *Canidae*. Several attempts to obtain an *in vitro* misfolded dog PrP by Protein Misfolding Cyclic Amplification (PMCA) [40] using a large number of prion strains and extensive PrP sequence comparison led to the identification of a single amino acid residue that may be responsible for its low misfolding proneness. Generation of transgenic mice over expressing mouse PrP with the identified substitution from dog PrP confirmed the implication of this amino acid residue *in vivo*. These transgenic mice showed complete resistance to TSEs after intracerebral inoculation with several mouse-adapted prion strains, corroborating the importance of certain key amino acid substitutions on TSE susceptibility. These results, besides helping to understand the mechanisms behind prion disease susceptibility and interspecies transmission barriers, may be useful for the design of new dominant negative PrPs able to block prion propagation and result in new therapeutic approaches for prion diseases [41].

## Results

### Dog PrP, a difficult protein to misfold *in vitro*

**Fig 1:**
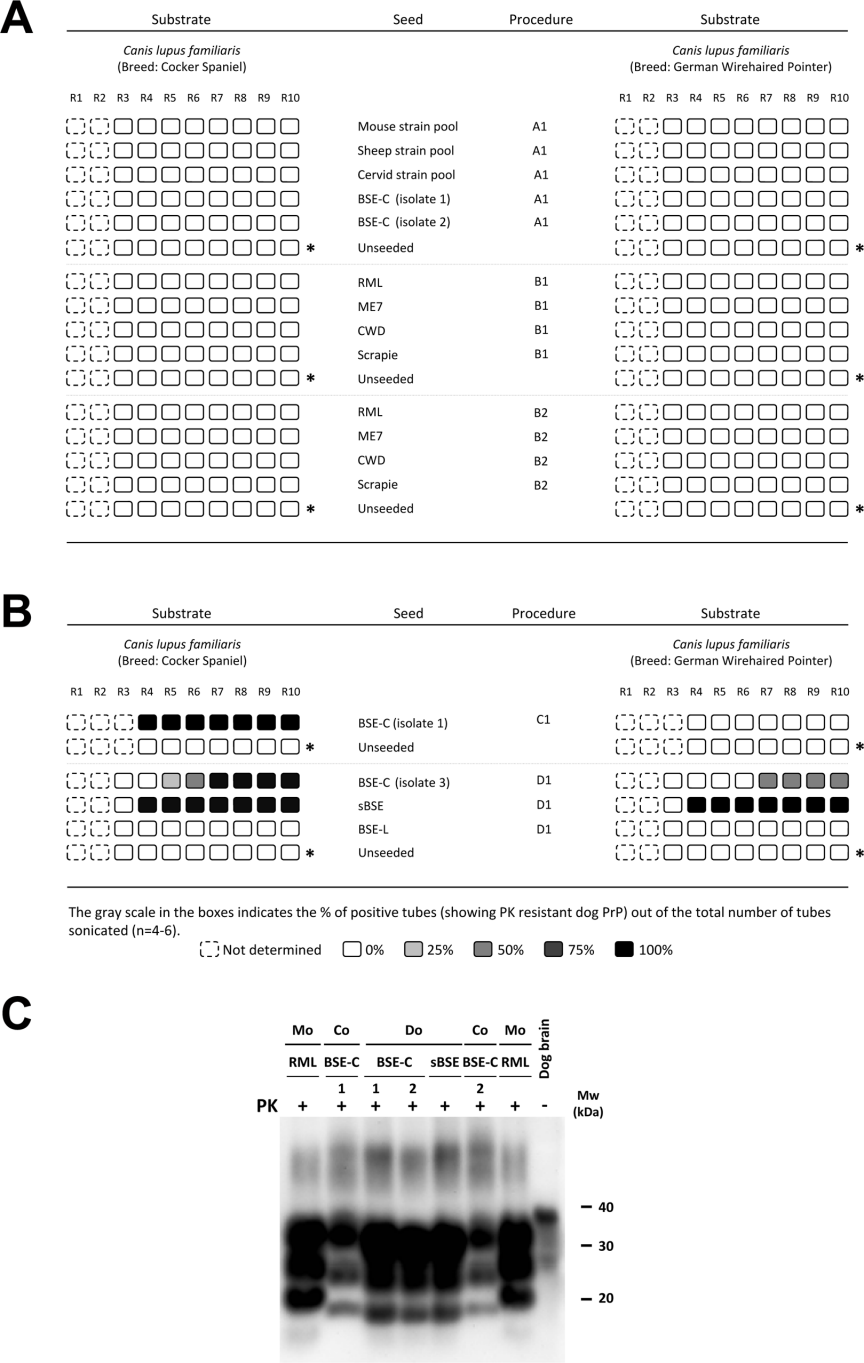
*In vitro* propagation experiments. **A & B**. Rounds (R1-R10) of serial PMCA using brain homogenates from two different breeds of dog as substrates: Cocker Spaniel and German Wirehaired Pointer. Several procedures to propagate different inocula over dog species were tested. *A1*: Standard 1–10% seeded PMCA with serial 1:10 dilution rounds. *B1*: 30–50% seeded PMCA with serial 1:10 dilution rounds. *B2*: same as B1 with the addition of zirconia-silica beads [52]. *C1*: 10% seeded PMCA with serial 1:2 dilution rounds. *D1*: 30–50% seeded PMCA with serial 1:2 dilution rounds. Different inocula or mix of inocula were used as seed. *Mouse strain pool*: Pool of mouse PrP^Sc^ strains containing equal amounts of ME7, RML, 22F, 22L, 87V, 22A, 79A, 139A and other spontaneously *in vitro* obtained strains. *Sheep strain pool*: Pool of sheep PrP^Sc^ strains containing equal amounts of 7 different scrapie isolates. *Cervid strain pool*: Pool of cervid PrP^Sc^ strains containing equal amounts of 2 mule deer and 2 elk isolates. *BSE-C*: Three different isolates of classical BSE (isolate 1, isolate 2 and isolate 3). *BSE-L*: Atypical L-type BSE. *sBSE*: Sheep BSE. * Twelve unseeded tubes were extended to 20 rounds for each set of experiments. Dog (Cocker Spaniel)-BSE obtained after the propagation of BSE-C (isolate 3) was shown in Vidal *et al*. 2013 [42]. **C**. One tube of round 10 of each PK-resistant sample was selected to show the biochemical analysis of BSE-C (1; isolate 1 and 2; isolate 2) and sheep BSE (sBSE) seeded material generated by PMCA compared to brain-derived BSE-C and RML. Samples were digested with 85 μg/ml PK and analyzed by Western blot using monoclonal antibody D18 (1:5,000). PK: Protease-K. Mo: Mouse. Co: Cow. Do: Dog. Dog brain: negative control; undigested dog whole brain homogenate. Mw: Molecular weight.

In order to evaluate the *in vitro* misfolding ability of dog PrP^C^, comparative *in vitro* seeding studies were performed using brain homogenates of two different breeds (Cocker Spaniel and German Wirehaired Pointer). Four distinct procedures with modifications on the propagation conditions and six prion strains of diverse origins (from mouse, sheep, cattle and deer) were used as individual or pooled seeds. After 10 serial PMCA rounds no misfolded dog PrP was detected in any case except when seeded with classical BSE (BSE-C), or a derivative of this one such as sheep BSE (Fig 1). Classical BSE and sheep BSE were the only seeds able to misfold dog PrP^C^ but requiring a modified propagation protocol (Fig 1B). To make sure that the signals observed correspond to real propagation and not to the remnant signal of the original seed or to the conversion of bovine PrP^C^ present in the brain homogenate of the seed, additional experiments were performed (S6 Fig). These results clearly suggest that dog PrP^C^ shows a very high resistance to be misfolded, despite the fact that it could be forced to misfold *in vitro* resulting in a protein with the biochemical characteristics, such as PK resistance and electrophoretic migration pattern, expected for a prion (Fig 1C) and with conserved biological features with respect to the original seed [42].

A comparison of PrP^C^ expression levels in the brains of different species (dog, rabbit, cat and mouse) did not show any significant differences between them. Thus, the possibility of a low expression level of PrP^C^ in dog brain being the responsible for its low misfolding proneness *in vitro* could be ruled out (S1 Fig).

### Searching for a clue in the primary sequence of PrP

**Fig 2:**
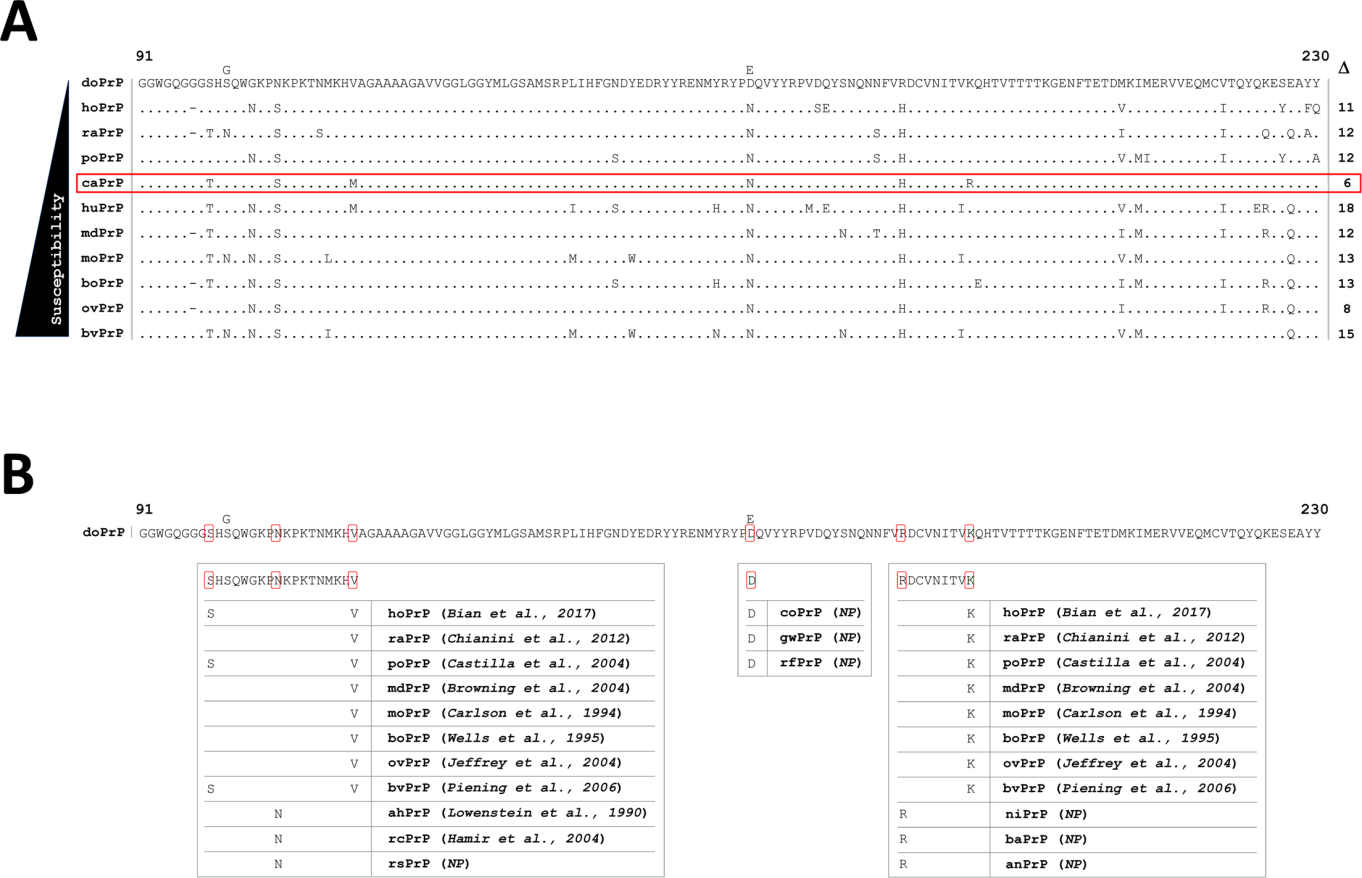
Dog PrP sequence alignments. **A**. PrP amino acid alignment based on residues 91–230 (dog PrP numbering) from species with different experimental and/or natural susceptibilities to prion diseases. Classification criteria were based on the number of representative prion strains able to be transmitted and cause disease in the host. Identical amino acids are indicated by dots. Δ: number of different amino acids compared to dog PrP. Note the similarity of the amino acid sequence in the cat (highlighted in red), a species known to be susceptible to naturally acquired prion disease. **B**. Upper line, dog amino acid residues 91–230. The 6 amino acid differences compared to cat PrP sequence are highlighted by red squares. Boxes show each highlighted amino acid and representative species in which the particular amino acid is present also. Where prion susceptibility of the species has been proven the reference is provided. NP: susceptibility not proven. Positions 101 and 163 are polymorphic in dogs (Ser/Gly and Asp/Glu, respectively). Species codification and accession numbers: doPrP, dog PrP (FJ870767.1); hoPrP, horse PrP (ACG59277); raPrP, rabbit PrP (NP001075490); poPrP, porcine PrP (AAA92862.1); caPrP, cat PrP (ACB97675.1); huPrP, human PrP (NP001073592); mdPrP, mule deer PrP (AY330343.1); moPrP, mouse PrP (NP035300); boPrP, bovine PrP (ABR92636.1); ovPrP, ovine PrP (NP001009481.1); bvPrP, bank vole PrP (AAL57231.1); ahPrP, armenian hamster PrP (AAA37014); rcPrP, raccoon PrP (ACA50738.1); rsPrP, red squirrel PrP (AAN16491); coPrP, coyote PrP (AGA63673); gwPrP, grey wolf PrP (AGA63687); rfPrP, red fox PrP (ACA50742); huPrP; human PrP (U29185.1); niPrP, nilgai PrP (AAV30507); baPrP, California big-eared bat (AAN16503); anPrP, anteater PrP (AAN16516).

An amino acid sequence comparable with dog PrP from a mammalian species with a different level of susceptibility to prion infection was identified (Fig 2A). The region 91–230 of the PrP sequence with the greatest similarity is cat PrP with just 6 distinct amino acids different. As felids are susceptible to infection with several different prion strains (e.g. BSE, CWD and CJD) [43–47], the 6 identified amino acid residues present in dog PrP in other species were examined in correlation with their reported susceptibility to TSEs (Fig 2B). From the initial six residues, just two, D163 and R180, were virtually exclusive to canids or present in uncommon mammalian species in which no prion disease has been reported; the nilgai (*Boselaphus tragocamelus*), Californian big-eared bat (*Corynorhinus townsendii*) and anteater (*Myrmecophaga tridactyla*). The other 4 amino acids differing between canine and feline PrPs also appear in species shown to be susceptible to prion infection (Fig 2B).

Among canids, dogs showed two polymorphisms in positions 101 and 163 of PrP; Ser/Gly and Asp/Glu, respectively. While the polymorphism in position 101 appears in different canine species, the Asp/Glu163 has been described in domestic dogs only, probably as a consequence of intensive selective breeding during the development of distinct breeds (S2 Fig). For position 163 there are only three mammalian species, besides canids, known to have an amino acid different to Asn. Two of them, pine marten (*Martes martes*) and wolverine (*Gulo gulo*), belong to the *Mustelidae* family and curiously enough have the same amino acid residue as the canids (S3 Fig). A representation of the phylogenetic tree could explain the possible origin of this polymorphism (S3B and S3C Fig). One other mammalian species is known to have an amino acid different to Asn or to Asp/Glu at position 163, the Squirrel monkey (*Saimiri sciureus*), which can have a Ser residue and is known for its susceptibility to prion diseases [48–50].

### An amino acid substitution caused significant changes in the surface of the protein

**Fig 3:**
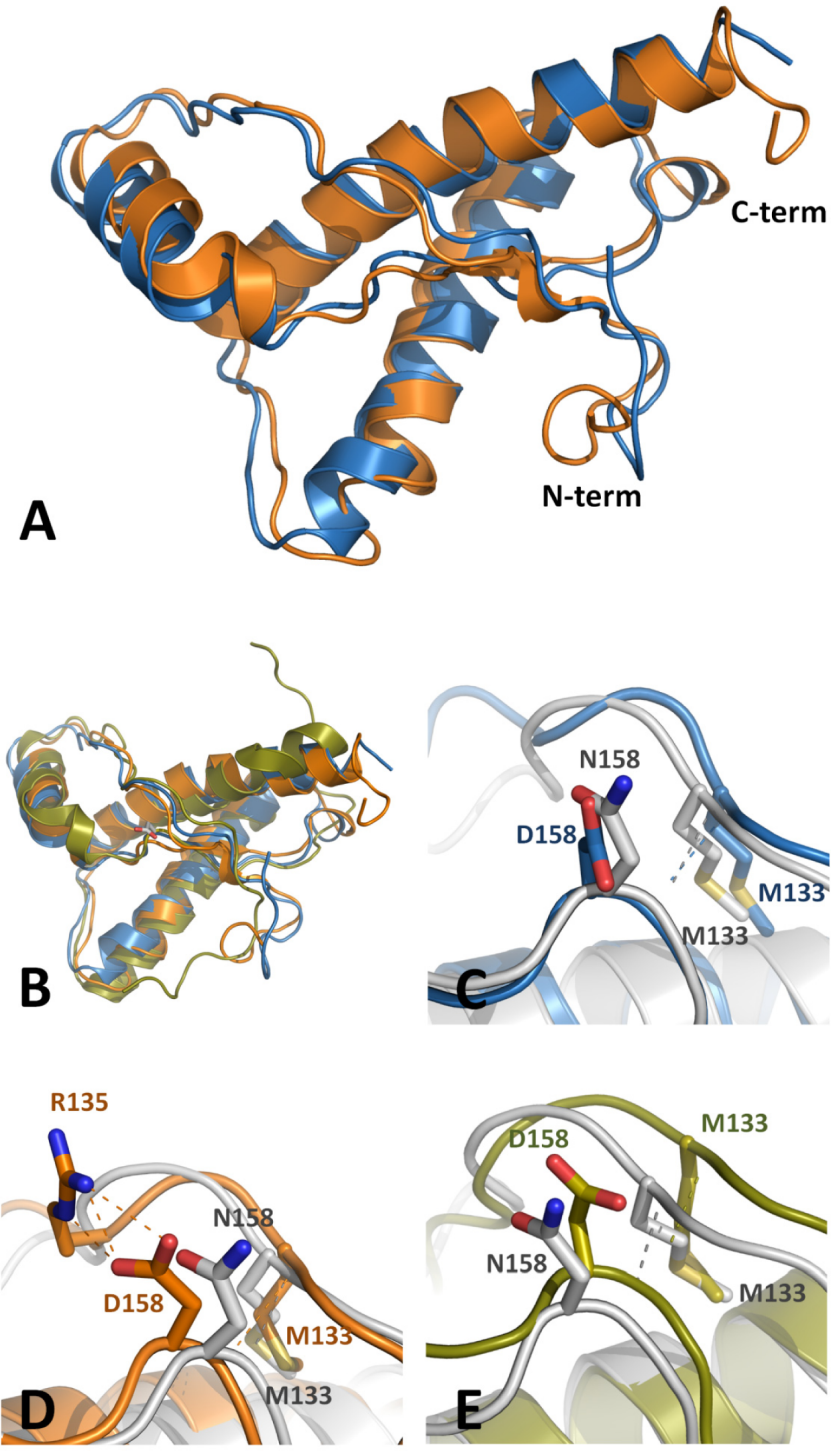
Structural models. **A.** Structural Cα backbone superposition of Model01 (blue) and Model02 (orange) cartoons (representing the mouse). Both N- and C-term regions are labeled. No significant structural rearrangements between the backbones can be observed. **B.** Structural Cα backbone superposition of canine prion protein (green) (PDB ID 1XYK) and Model01 (blue) and Model02 (orange) cartoons. Despite some detectable differences, overall folding of the models with respect to dog PrP remains similar. **C-E.** Local environment of mouse Asn158 (N158) from PDB ID 1XYX (gray) and arrangement of Asp158 (D158) **(C)** (PDB ID 1XYK- blue), Asp158 (D158) from Model01 **(D)** (orange) and Asp158 (D158) structure in Model02 **(E)** (green). All residues are labeled and represented as sticks. N158 from mouse PrP shows a hydrogen bond to M133 stabilizing the Cα backbone. While D158 from Model02 does not establish other interactions, D158 conformer from Model01 exhibits a change leading to the formation of a salt bridge with R135. Analogous hydrogen bond can be observed between N158 and R135 also in mouse PrP but, no similar interaction can be found with surrounding residues in dog PrP.

To determine if the presence of the particular amino acid residue present in canids affects the structural arrangement of PrP from a TSE susceptible species an *in silico* structural analysis was performed. Two structural models based on mouse prion protein were generated which contained the apparently critical canine substitution (N158D, equivalent to position 163 in dog PrP) using as templates the averaged NMR structure from PDB ID 2L39 (Model01) and X-ray crystallography structure from PDB ID 4MA7 (Model02). Cα superposition of Model01 onto Model02 overlays 103 residues (out of 114 in Model01) with a root-mean-square deviation (R.M.S.D.) = 1,420 Å, showing no significant structural rearrangements in the backbone between them (Fig 3A). Similarly, overall folding of the mutants is the same as those observed in the native mouse PrP structures used as templates. Cα backbone superposition of Model01 onto PDB ID 2L39 overlays 105 residues (out of 114 in Model01) with an R.M.S.D. = 0,396 Å and Cα superposition of Model02 onto PDB ID 4MA7 overlays 106 residues (out of 110 in Model02) with an R.M.S.D. = 0,055 Å.

Superposition of both models Cα backbone onto canine prion protein (PDB ID 1XYK) show some differences although overall folding remains similar (Fig 3B). Model01 onto canine prion protein overlays 93 residues (out of 114 in Model01) with an R.M.S.D. = 2,052 Å and Cα superposition of Model02 overlays 97 residues (out of 110 in Model02) with an R.M.S.D. = 2,170 Å.

A closer analysis of the residue Asn158 (N158) from mouse PrP in all reported structures and also both models, show a conserved hydrogen bond to Met133 (M133) stabilizing the Cα backbone and different conformations in the prion surface. Nevertheless, while Asp158 (D158) from Model02 does not establish other interactions with surrounding residues (Fig 3C), D158 conformer from Model01 exhibits a change in the side-chain conformation leading to the formation of a salt bridge interaction with Arg135 (R135) side chain (Fig 3D) at the loop linking strand-β1 and helix α1.

Analogous hydrogen bond can be observed between N158 and R135 also in mouse PrP PDB ID 2L39 structure but, surprisingly, no similar interaction can be found with surrounding residues in Canine-PrP (PDB ID 1XYK) (Fig 3E).

Analysis of the local environment at N158/D158, when reported prion structures are compared, shows slight mobility in residue 158 but a higher one on R135, suggesting that the R135-D158 interaction depends mainly on the R135 side chain rotamer which is comparable with those observed in wild-type mouse prion structures (S4 Fig).

**Fig 4:**
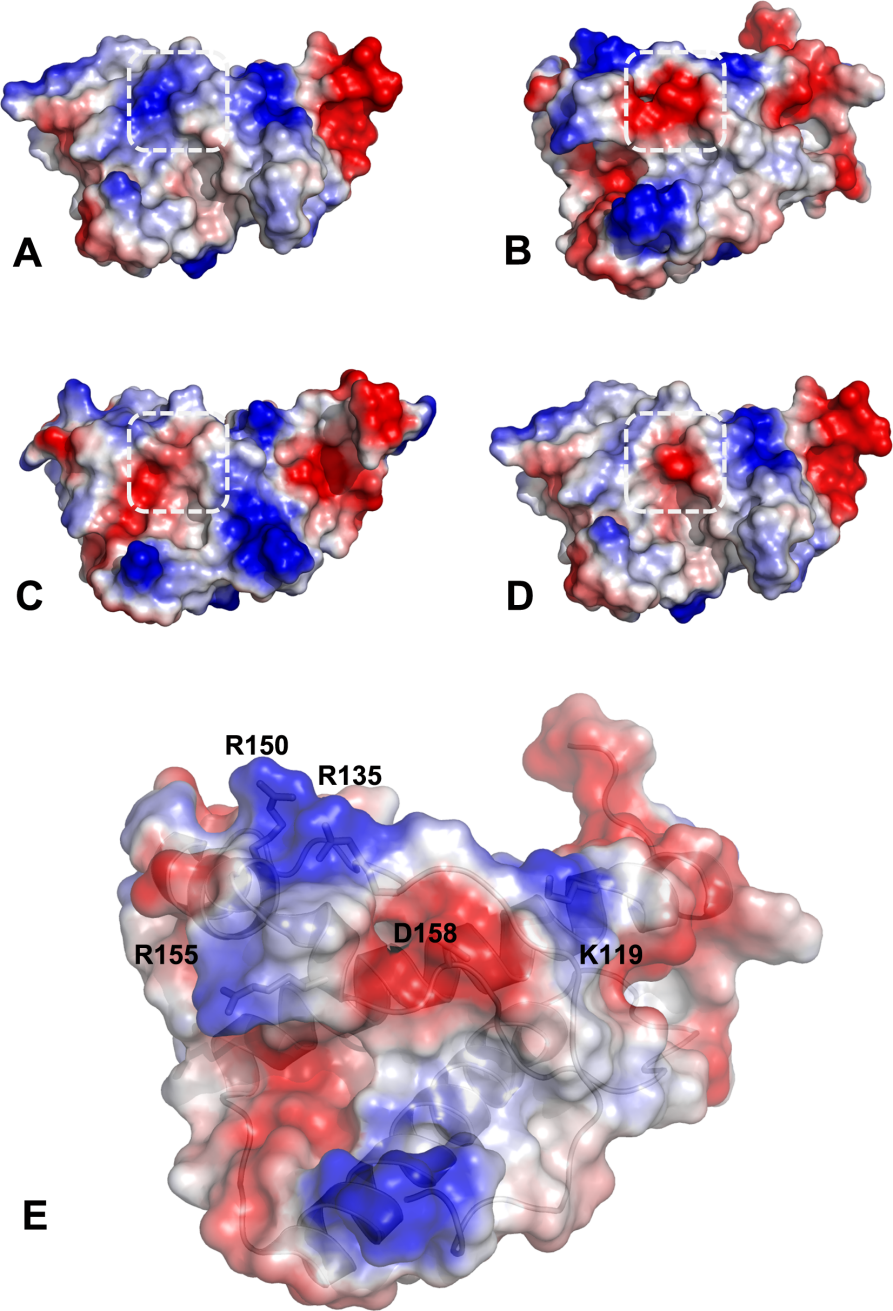
Electrostatic potentials on the surface of protein structures. **A.** Wild-type mouse prion protein (PDB ID: 4MA7). **B.** Canine prion protein (PDB ID: 1XYK). **C-E.** Modelled structures: Model01 **(C)**, Model02 **(D)**, and distribution of positively charged residues surrounding Asp158 in wild-type canine prion protein **(E)**. Acidic regions are colored in red and basic regions in blue, residue 158 for all structures is highlighted in dashed line white box. Amino acid D158 located within an area with four Arg/Lys residues (R135, R150, R155 and K119) introduces a positively charged residue in that region, in dog PrP, Model01 and Model02 changing the local charge distribution compared to mouse wild-type PrP.

The most notable change due to the introduction of D158 was detected through the study of electrostatic potentials on the surface of PrP which revealed larger differences when polar amino acid D158 was situated within an area with four Arg/Lys residues (Fig 4A), Arg135 (R135), Arg150 (R150), Arg155 (R155) and Lys119 (K119). Acidic properties of D158 in canine PrP (Fig 4B) introduces a positively charged residue in that region, as occurs in Model01 (Fig 4C) and Model02 (Fig 4D), changing the local charge distribution of mouse wild-type PrP (Fig 4E).

### *In vitro* studies to evaluate the misfolding properties of mouse PrPs containing the selected dog amino acid residue substitutions

**Fig 5:**
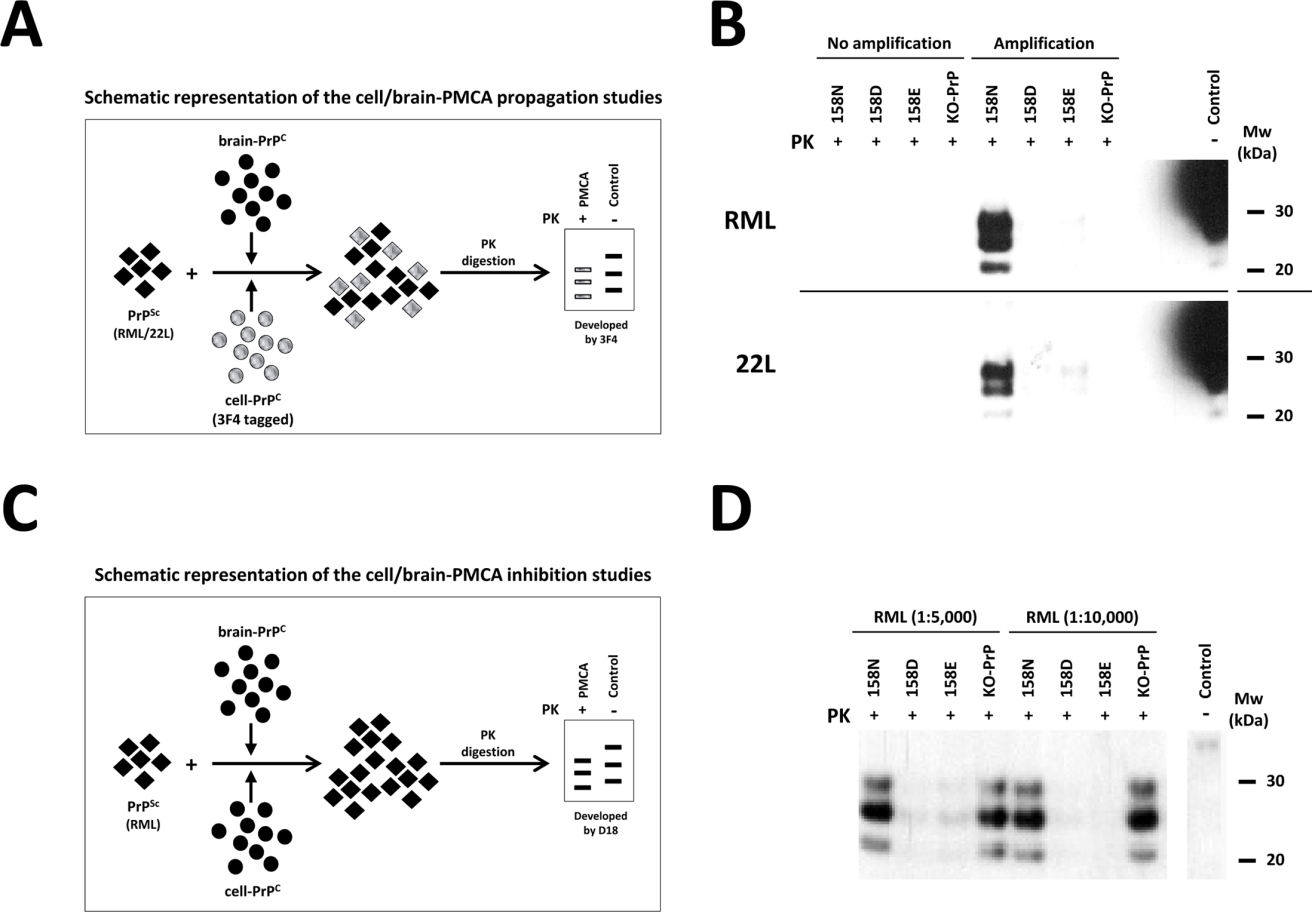
Two cell/brain-PMCA methodologies to study the effect of the residue 158 in mouse PrP. **A**. Schematic representation of the cell/brain-PMCA propagation study. Brain-derived PrP^C^ (black filled circles) is mixed with cell-derived 3F4-tagged PrP^C^ (grey filled circles) and seeded with PrP^Sc^ (RML or 22L, black filled squares). The resulting 3F4-tagged PrP^Sc^ (grey filled squares) is specifically detected by the 3F4 antibody. **B**. A 1:40 dilution of RML or 22L were used as seeds for a PMCA based on mouse brain homogenate mixed with cellular 3F4-tagged substrates containing mouse PrP N158, D158, E158 or without PrP (*PRNP*^*0/0*^; KO-PrP). Non-PMCA amplified samples and samples subjected to one 24 h single round of PMCA were digested with PK (20 μg/ml) and analyzed by Western blot using monoclonal antibody 3F4 (1:10,000). Control: undigested human brain homogenate. **C**. Schematic representation of the cell/brain-PMCA inhibition studies. Brain derived PrP^C^ (black filled circles) is mixed with cell derived PrP^C^ (black filled circles) and seeded with PrP^Sc^ (RML, black filled squares). Total resulting PrP^Sc^ is detected by antibody D18. **D**. 1:5,000 or 1:10,000 dilutions of RML were used as seeds for a PMCA based on mouse brain homogenate mixed with cellular substrates containing mouse PrP N158, D158, E158 or without PrP KO-PrP. Samples subjected to one 24 h single round of PMCA were digested with PK (20 μg/ml) and detected by the monoclonal antibody D18 (1:10,000). Both procedures showed a significant inhibitory effect of the D158 and E158 substitutions over the *in vitro* propagation of RML/22L mouse prion strains. Control: undigested mouse brain homogenate. Mw: Molecular weight.

To further explore the findings presented above, this study focused on amino acid residue 163 (dog PrP numbering) and how the presence of an Asp/Glu instead of an Asn could affect the ability of certain PrPs to misfold *in vitro*. Mouse PrP was chosen as a model and genetic constructs were prepared to produce mouse PrP with either Asn (N158 in mouse PrP numbering), Asp (D158) or Glu (E158) in cells. In all the cases, expression level of mutated PrPs in cell cultures was checked by Western blotting to make sure that the relative ratios of cell PrP^C^ versus brain PrP^C^ were between 1:1 and 1:3, being the cell PrP amount equal or minor than the brain PrP amount in all the experiments (see an example of cell PrP expression levels in S7 Fig). The three proteins were subjected to two types of study based on cell-PMCA. The first type of cell-PMCA evaluated the *in vitro* propagation ability of each of the proteins that, besides the mutation in position 158, contained a 3F4-tag. These proteins mixed with mouse whole brain homogenates (mouse PrP^C^ does not contain the 3F4 epitope) were subjected to a single cell-PMCA round seeded with two different mouse prions (RML and 22L) (Fig 5A). Regardless of the seed used, only the N158 protein was able to misfold *in vitro*, unlike proteins E158 and D158 (Fig 5B). The second study evaluated the inhibitory capacity shown by each of the three proteins over the misfolding of wild-type mouse PrP^C^ (Fig 5C). In this case, RML was used as seed for a single cell-PMCA round in which the inhibitory effect of proteins D158 and E158 on the propagation of wild-type mouse PrP was clearly demonstrated (Fig 5D).

Both experiments suggested that D158 and E158 substitutions impede the misfolding of mouse PrP as well as inhibited the propagation of wild-type mouse PrP.

### Inability of transgenic N158D mouse PrP brains to propagate prions *in vitro*

The previous results, obtained *in vitro*, led to the generation of a new transgenic mouse model expressing mouse PrP with the substitution N158D. Two hemizygous transgenic mouse lines were generated, Tg402 and Tg403 with a *PRNP*^*0/0*^ background and with transgene expression levels of 1X and 2X, respectively compared to wild-type mouse endogenous PrP^C^ levels (S5 Fig). In order to demonstrate that the mutant PrP is properly expressed in the transgenic animals, a comparative immunohistochemistry was performed in wild-type and transgenic mouse lines. A fine granular neuropil immunolabeling (corresponding to PrP^C^ on the dendrite cell membrane) and absence of labeling within the pericarion were observed (S8 Fig). Six hundred days old animals of both transgenic lines had a normal phenotype with no neurological clinical signs.

**Fig 6:**
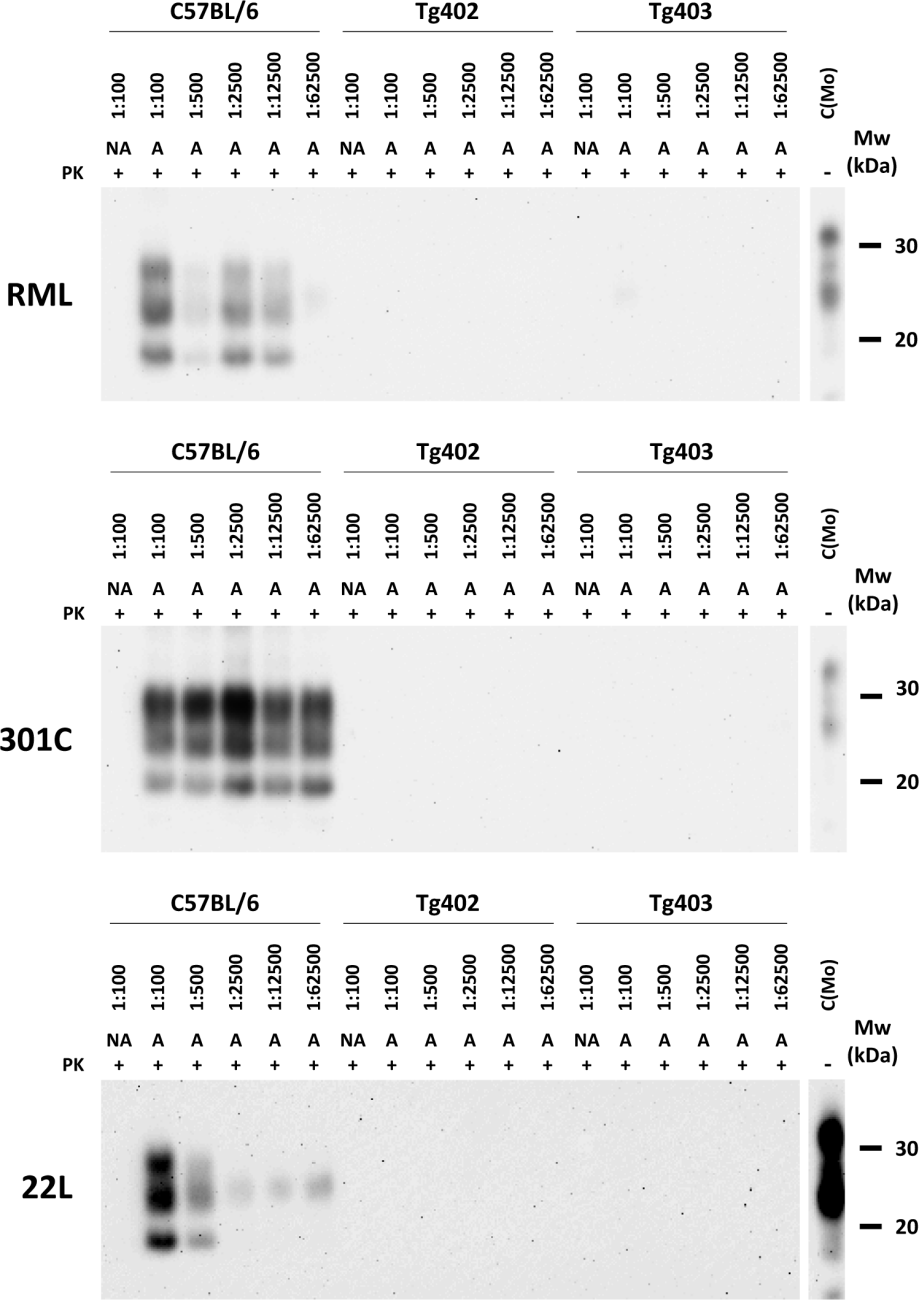
*In vitro* propagation ability of RML, 301C and 22L mouse prion strains by PMCA. 10% brain homogenates from transgenic N158D PrP mouse lines (Tg402 and Tg403) were seeded with the different mouse prion strains (RML, 301C and 22L) at the indicated dilutions and subjected to one single 48h PMCA round. All samples [non-PMCA amplified (NA) and amplified samples (A)] were digested with 85 μg/ml of protease-K (PK) and were analyzed by Western blot using SAF-83 (1:400) monoclonal antibody. No mouse prion strains were able to propagate in any of the transgenic N158D PrP mouse brain homogenates. C(Mo): undigested mouse brain homogenate. Mw: Molecular weight.

Brain homogenates from animals of both lines were used to evaluate their capacity to propagate mouse prions *in vitro*. Three different mouse prion strains (RML, 301C and 22L) were used as seeds for PMCA using the brain homogenates of the two transgenic lines as substrate and a brain homogenate of wild-type C57/BL6 mouse as positive propagation control. None of the brain homogenates containing D158 PrP was able to propagate any of the mouse prions used (Fig 6).

### Prion resistance of the transgenic mice expressing the N158D mouse PrP to prion challenge

**Fig 7:**
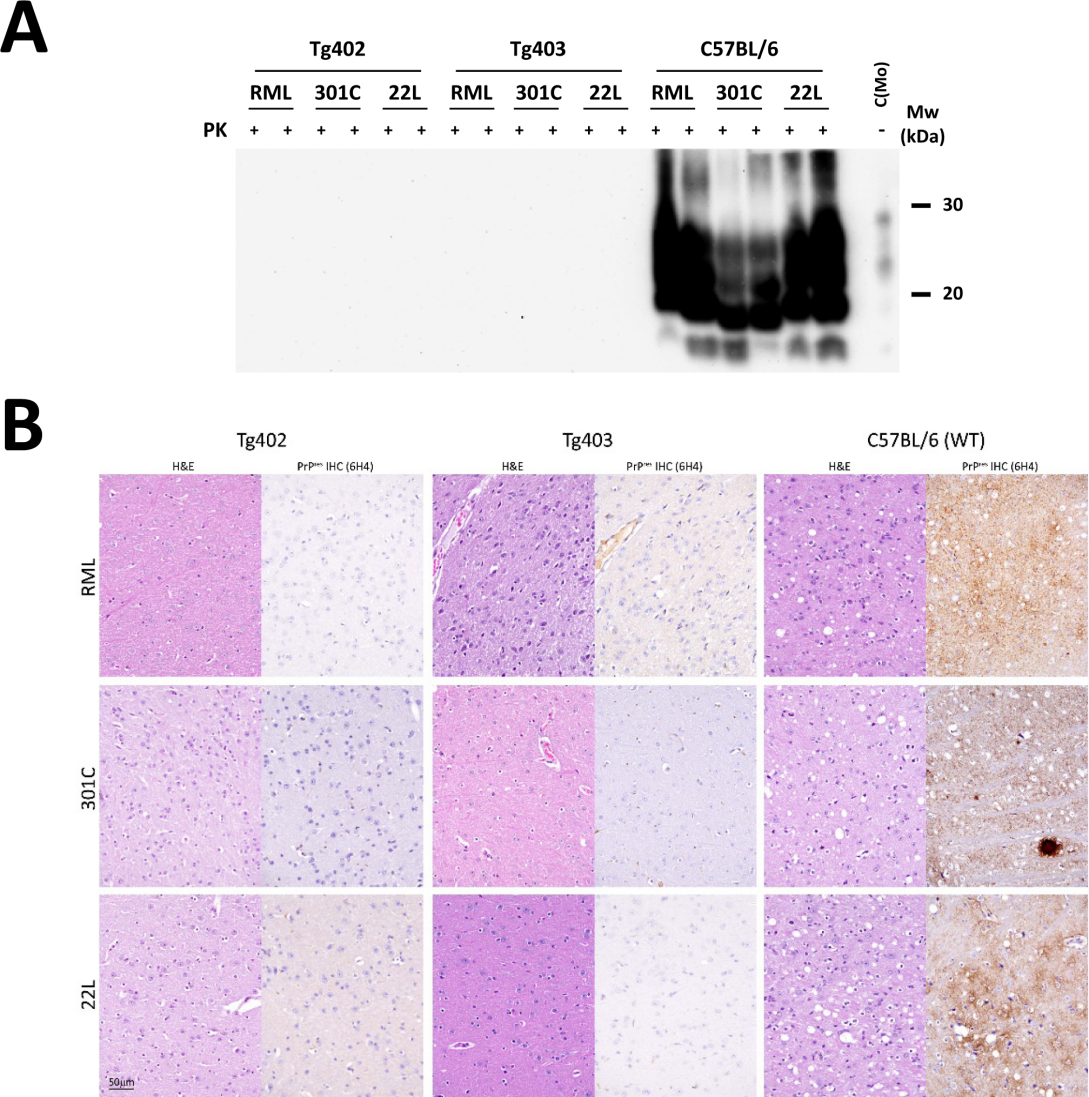
Brain analyses from RML, 301C and 22L inoculated mice. **A**. Two mouse brains from each inoculated group (Tg402, Tg403 and C57BL/6) were selected to determine the presence of protease-K (PK) resistant PrP. Samples were treated with 85 μg/ml of PK and protease resistant protein was analyzed by Western blot using SAF-83 (1:400) monoclonal antibody. Only C57BL/6 inoculated brain samples showed characteristic PK-resistant PrP migration patterns. All transgenic mouse brains were devoid of all PK-resistant PrP. C(Mo): undigested mouse brain homogenate. **B**. Histopathological characterization of different mouse prion strains (RML, 301C and 22L) inoculated intracerebrally in Tg402, Tg403 and C57BL/6 wild-type mice. Two mouse-adapted scrapie derived strains (RML and 22L) and a mouse-adapted BSE derived strain (301C) showed typical spongiform change only in the brains of wild-type mice in hematoxylin and eosin (H&E) stained sections. No TSE-related lesions were observed in Tg402 nor in Tg403 inoculated mice with any of the strains. Similarly, upon immunohistochemical labeling with 6H4 antibody against prion protein [PrP^res^ IHC (6H4)] (1:1,000), only the wild-type mice had immunolabeled deposits of prion protein. All images were taken at the same magnification in the region of the diencephalon. WT: Wild-type. Bar 50 μm.

Animals from both transgenic lines, together with C57/BL6 wild-type animals as TSE susceptible positive controls, were inoculated with three different mouse-adapted prion strains (RML, 301C and 22L). While the wild-type mice succumbed to each prion disorder with the expected incubation times for each strain (Table 1), none of the transgenic animals showed any clinical signs even after 550 days post-inoculation (dpi). Biochemical analysis of the brains of inoculated transgenic animals confirmed the absence of PK-resistant PrP (PrP^res^) depositions, while the infected C57/BL6 brains showed high amounts of PrP^res^ with electrophoretic migration patterns characteristic of each of the strains used for inoculation (Fig 7).

**Table 1:** Inoculation of mouse-derived prion strains into Tg402, Tg403 and C57BL/6 mice.

Model	Inoculum	Survival time (dpi) (±SEM)^a^	Attack rate^b^
Tg402	RML	>550^c^^,^^d^	0/12 (0%)
	301C	>550^c^^,^^e^	0/13 (0%)
	22L	>600^c^^,^^f^	0/10 (0%)
	Non-inoculated	>600^c^	0/20 (0%)
Tg403	RML	>550^c^^,^^g^	0/11 (0%)
	301C	>550^c^	0/9 (0%)
	22L	>550^c^	0/9 (0%)
	Non-inoculated	>600^c^	0/20 (0%)
C57/BL6	RML	168 (±1)	8/8 (100%)
	301C	213 (±4)	10/10 (100%)
	22L	148 (±5)	7/7 (100%)
	Non-inoculated	>600^c^	0/20 (0%)

Inconclusive and negative results were confirmed by using more than one disease-associated PrP (PrP^d^) detection method.

^a^ SEM: Standard Error of the Mean. Not indicated when the whole group was sacrificed at the same day post inoculation (dpi).

^b^ Animals were considered TSE positive when spongiform lesions and/or PrP^d^ was detected, either through immunohistoche-mistry (IHC) or Western blotting (WB).

^c^ All animals were TSE negative and humanely euthanized due to non-TSE causes.

^d^ Two animals death from intercurrent diseases at 235 and 513 dpi.

^e^ One animal death from intercurrent diseases at 338 dpi.

^f^ One animal death from intercurrent diseases at 470 dpi.

^g^ Two animals death from intercurrent diseases at 272 and 508 dpi.

Histopathological characterization of the brains confirmed the presence of typical TSE related neuropathological findings, i.e. spongiform change and PrP immunolabeled deposits, only in wild-type mice but not in Tg402 or Tg403 mice brains (Fig 7B).

## Discussion

To establish TSE resistance in any given species requires that several features are examined before a definitive conclusion can be reached. Since TSEs are a group of neurodegenerative disorders, sometimes with extremely low prevalence, the absence of TSE cases reported for certain species may be due to reduced size populations or because sporadic deaths in wildlife species are rarely investigated. For example, sporadic BSE cases in cattle affect around 2–5 individuals per million per year and were only detected when millions of animals were analyzed as a consequence of the “mad cow” crisis [51]. Therefore, to determine the susceptibility of certain species to TSEs experimental inoculations in a statistically representative number of individuals are required. Performing experimental infections in some mammalian species is relatively easy but for others difficult or even impossible due to a number of technical and ethical/cultural hindrances, and the latter is the reason of the absence of literature reporting prion inoculations in dogs. Although there are reports of suspected TSE cases in canids [35–37], none of the results were conclusive and there is no definitive proof of prion disease in this genus. BSE contaminated feed was undoubtedly fed to several wild canine species in UK zoos and domestic dogs during the BSE epidemic which suggests that, if not completely resistant, canids have a very low susceptibility to prion infection, as many other mammals in zoological collections fed contaminated food succumbed to the disease [7].

Given the lack of rigorous experimental challenge studies in dogs, the definitive proof of their TSE resistance may be derived by inoculation of experimental transgenic mice models expressing dog PrP (ongoing experiments). However, based on the *in vitro* results presented here, including several attempts to misfold dog PrP by PMCA, it is reasonable to assume that dog PrP is highly resistant to misfolding as the same *in vitro* methodology misfolds the PrP of species previously considered to be TSE resistant such as the rabbit [13].

Dog PrP could eventually be misfolded *in vitro* but using only BSE and BSE-derived strains, which retained their ability to infect bovine PrP expressing transgenic mice [42]. This is strong evidence of the powerful misfolding capacity of PMCA and endorses the use of this system to evaluate the misfolding ability of different species’ PrPs. However, regarding the evaluation of the degree of transmission barrier, PMCA is qualitative or semi-quantitative at best. In order to semi-quantify the strength of an interspecies transmission barrier, the *in vitro* process should be done in a controlled manner since ultimately PMCA might convert any mammalian PrP through the seeding with any kind of prion inocula if enough rounds during the *in vitro* process are performed. The number of cycles in each round and the number of rounds are empirical data and should be established by comparison with an standard [52]. In this case, the degree of canine PrP resistance to misfolding was not specifically evaluated in comparison to other species. Nonetheless, a low susceptibility to misfolding was concluded due to the requirement of modified PMCA conditions which increased the possibility of misfolding and because from the seeds tested just BSE and derived strains were able to induce misfolding. These issues were not observed when rabbit PrP was misfolded *in vitro*, although its low susceptibility to prion infection *in vivo* is well known [13].

Assuming a high but not complete resistance of dog PrP to misfolding, we focused on the identification of the amino acids and their positions in the prion protein that could be responsible. A PrP sequence alignment with species phylogenetically close to canids showed that feline PrP was the most similar in terms of primary structure. Cats are highly susceptible to three distinct prion strains (BSE, CWD and CJD) [43–47] and this led to the comparison of a small number of differing residues with PrPs from other species. From these comparisons, three specific amino acids were identified, although one of them was readily discarded due to its presence in PrPs of TSE susceptible animals. Of the other two identified residues detected in canine PrP, the Asp/Glu in position 163 was chosen as the most relevant for canine resistance. The presence of an Asn in that position is a highly conserved residue in mammalian PrP sequences from different species and located in the loop of residues linking helix-α1 and strand-β2. Therefore, from all the PrP^C^ structures described (i.e. human, mouse, rabbit, sheep, cow, horse, hamster, cat, bank vole, pig and elk [23, 24, 53–60]), only the structural model of canine wild-type PrP [24] allows studying the role of Asp/Glu polymorphism in the overall structure of PrP. When N158 is replaced by D158 in the mouse PrP, no structural changes in the Cα backbone at the mutated position are observed in our *in silico* models except those stabilizing the backbone. This is probably due to D158 (D163 in dog PrP numbering) being highly exposed to the solvent and like most charged residues in proteins, it could play a role in the folding of the protein. A hydrogen bond present between N158 and R135 is not present in canine PrP (between residues D163 and R140 in dog PrP numbering). Nevertheless, a salt bridge could be established between D158 and R135 as observed in Model01 due to the high mobility of the R135 side chain present in mouse prion structures. Both non-covalent interactions are relatively weak but the presumptive salt bridge R135-D158 could contribute to D158-mouse PrP and canine-PrP overall increased structural stability.

The definitive selection of this substitution as a candidate for prion resistance came from its role on the surface of the PrP molecule and its influence over the basic area comprised of R135, R150, R155 and K119. Assuming these residues participate in the PrP conversion to the disease associated isoform, D158 probably disturbs that PrP region as well as establishing a salt bridge with R135 limiting the latter’s role in conversion. This is supported by recent findings in transgenic *Drosophila* expressing mouse PrP with N158D substitution where it impairs the locomotor dysfunction developed when wild-type mouse PrP is expressed [61].

Taking advantage of the PMCA system that allowed examining if this amino acid could cause a significant change in the misfolding and propagation ability of known prion strains, two assays were performed. Based on the expression of mouse PrP with the desired N158D or N158E substitutions (equivalent to position 163 in dog PrP numbering) in cell cultures from neuronal origin, cell-PMCAs were performed to test their misfolding ability. Both studies showed clearly that the presence of an Asp or a Glu in position 158 of the PrP significantly hindered its misfolding propagation ability. Therefore, as predicted by surface charge distribution analysis, the presence of a negatively charged amino acid (Asp or Glu) is needed for a significant alteration in misfolding ability. As well as being unable to misfold, these mutants were able to block the propagation over wild-type mouse protein. This data highlights the potential for using these proteins as dominant negatives, blocking the propagation of prions, which has been a successful strategy for other dominant negative PrPs in cell cultures [62], and becoming an efficient anti-prion therapy. Although the mechanism by which this proteins block prion propagation over wild-type proteins is unknown, our data suggests that dominant negative proteins may act by binding prion seeds or misfolded proteins and out-competing the PrP that can be misfolded.

The results obtained in cell-PMCA experiments did not clearly distinguish which of the substitutions, N158D or N158E, gave rise to the PrP with lowest misfolding capacity as they showed almost identical behaviour. However, there was a faint PrP^res^ signal when 22L was used to seed N158E PrP propagation, suggesting that at least for certain strains, N158D substitution may exert a major blocking effect than N158E. Both are negatively charged residues with similar molecular weights, so a similar effect would be expected in PMCA for N158D and N158E mutants. Nonetheless, a classification based on the environment at protein structures for the 20 amino acids [63, 64] sets several groups for residues, in which both negatively charged residues show distinct features regarding their propensity to be involved on protein surfaces or in binding regions. Asp (D) shows similar tendency to both while Glu (E) shows a strong preference to be exposed to solvent on protein surfaces [65]. Analysis of electrostatic potentials on the surface for N158D PrP shows that it is located in a region surrounded by positively charged residues. Although the negative charge of both Asp (D) and Glu (E) suggests they could play a similar role in terms of surface charge distribution, the shorter side-chain of Asp (D) makes it more rigid within protein structures than Glu (E), with a larger and more flexible side-chain. Alterations in surface electrostatic potential due to the negatively charged residues could reduce PrP^c^ to PrP^Sc^ conversion efficiency, but also could affect the structure of the fibrils decreasing their formation. The rigidity of Asp (D) compared to Glu (E), could therefore explain the slight differences observed in the PMCA results with N158D and N158E PrPs. The replacement of N158 by Asp (D), with its restricted mobility, would possibly lead to a major disturbance of the surrounding area in order to accommodate the negative charge. In contrast, the larger side-chain of Glu (E) could allow the adoption of a wider range of conformers, possibly reducing the effect observed for 158D and allowing some conversion of N158E PrP in PMCA. Thus, we decided to choose Asp (D) instead of Glu (E) for the substitution in the PrP of the transgenic mouse model presented herein. Also taking into account that all the dog brain homogenates used in previous PMCA studies contained the D163 polymorphism. Thus, our results on the effect of D163 on the high resistance of canines to prion disorders would be applicable to some breeds of domestic dogs, although the similarities of Asp (D) and Glu (E) and the results obtained in brain-cell PMCA studies (Fig 5) indicate that it might be applicable also to the domestic dog breeds bearing E163 polymorphism (S2 Fig).

The expression levels of the canine PrP transgene in both mouse lines was close to PrP^C^ expression in the brain of wild-type animals, thus, the results obtained from their experimental challenges are unlikely to be affected due to over expression, which is known to accelerate prion disease development [66]. The three different mouse-adapted prion strains used (RML, 22L and 301C) did not cause clinical disease nor histological brain lesions in any of the challenged transgenic animals, indicating that this specific amino acid substitution prevented mouse PrP from misfolding *in vivo*, in agreement with the *in vitro* results. Additionally, the complete absence of PK resistant PrP, by Western blotting (WB) and immunohistochemistry (IHC), compared to the extensive PrP^res^ accumulation in wild-type mice inoculated with the same prion strains further supports the protective properties of this specific amino acid substitution. Despite the possible existence of subclinical carriers with minimal amounts of PrP^Sc^, undetectable by conventional methods (WB and IHC) among the inoculated transgenic mice, a significant delay in the disease development was clearly demonstrated.

Although the possibility of overcoming this polymorphic transmission barrier through serial *in vivo* passages cannot be ruled out from the results shown here, the high resistance observed to misfolding both *in vivo* and *in vitro* shows the significant effect of this substitution on reducing the misfolding proneness of the protein. Although performed in transgenic mice, these results probably explain the purported resistance canids have to TSEs despite having been exposed to infectious prions by consuming TSE affected animals such as sheep, cattle or cervids. Definitive proof of their resistance will require the generation of transgenic mice expressing whole dog PrP to allow the appropriate bioassays.

The potential dominant negative effect of the mutated protein presented in this work over the wild-type protein (ongoing study) may determine their future as anti-prion therapies.

## Materials and methods

### Inocula preparation for *in vivo* and *in vitro* prion propagation studies

Brain homogenates (10^−1^ in PBS) for use as seeds for PMCA or direct intracerebral inocula were prepared manually using a glazed mortar and pestle from brains of animals clinically affected by various TSEs: scrapie (six isolates), BSE-C (three isolates) and atypical L-type BSE field cases supplied by the Laboratorio Central de Veterinaria (Algete, Madrid, Spain); SSBP/1 (scrapie), ME7, 22F, 22L, 87V, 22A, 79A and 139A supplied by Animal and Plant Health Agency (APHA) (New Haw, Addlestone, Surrey, UK); CWD (2 mule deer and 2 elk isolates) supplied by the Department of Veterinary Sciences (Laramie, WY, USA); RML supplied by Rocky Mountain Laboratories (Hamilton, MT, USA) and sheep BSE supplied by Ecole Nationale Vétérinaire (Toulouse, France).

### *In vitro* propagation of prions by PMCA

*In vitro* prion propagation and PrP^res^ detection of amplified samples was performed as described previously with minor modifications [13, 67]. Briefly, two different dog breed (Cocker Spaniel and German Wirehaired Pointer) brains (provided by Veterinary Faculty from Autonomous University of Barcelona) used for substrates were perfused (immediately after euthanasia) using PBS + 5 mM EDTA and the blood-depleted brains were frozen immediately until required for preparing the 10% dog brain homogenates (PBS + NaCl 1% + 1% Triton X-100). 50–60 μl of 10% dog brain homogenates, either unseeded or seeded with the corresponding prion strain were loaded onto 0.2-ml PCR tubes and placed into a sonicating water bath at 37–38°C without shaking. Tubes were positioned on an adaptor placed on the plate holder of the sonicator (model S-700MPX, QSonica, Newtown, CT, USA) and subjected to different procedures of 24 h serial rounds of PMCA: A1: Standard 1–10% seeded PMCA with serial 1:10 dilution rounds. B1: 30–50% seeded PMCA with serial 1:10 dilution rounds. B2: same as B1 with the addition of 1 mm zirconia/silica beads (BioSpec Products), that have been shown to improve PrP^C^ to PrP^Sc^ conversion in PMCA [52]. C1: 10% seeded PMCA with serial 1:2 dilution rounds. D1: 30–50% seeded PMCA with serial 1:2 dilution rounds. All the samples were incubated in cycles of 30 min followed by a 20 s pulse of 150–220 watts sonication at 70–90% of amplitude. An equivalent number of unseeded (6–12 duplicates) tubes containing the corresponding brain substrate were subjected to the same number of rounds of PMCA in order to control cross-contamination and/or the generation of spontaneous PrP^res^. The detailed protocol for PMCA, including reagents, solutions and troubleshooting, has been published elsewhere [68].

### Biochemical characterization of *in vitro*- and *in vivo*-generated prion strains

*Protease resistance assay*: PMCA treated samples and 10% brain homogenates from prion inoculated mice were incubated with 85–200 μg/ml of Protease-K (PK) (Roche) for 1 h at 42°C with constant agitation at 450 rpm (Thermomixer comfort Eppendorf) as described previously [40]. Samples were mixed previously (1:1, v/v) with 10% Sarkosyl (Sigma-Aldrich) digestion buffer and the digestion was stopped by adding electrophoresis Laemmli buffer *NuPAGE* (Invitrogen Life Technologies).

*PK-resistant PrP detection*: Protein inmunodetection by Western blotting (WB) was performed after separating proteins by sodium dodecyl sulfate-polyacrylamide gel electrophoresis (SDS-PAGE). 4–12% NuPAGE Midi gels (Invitrogen Life Technologies) were used. The proteins were electroblotted onto nitrocellulose membranes (Protran BA85, GE Healthcare). Membranes were probed with mouse monoclonal antibody D18 (1:5,000–10,000), POM1 (1:10,000) or Saf83 (1:400) (Cayman Chemical) and visualized with horseradish peroxidase–conjugated seconday antibody and chemiluminescence using the Super Signal West Pico kit (Thermo Scientific Pierce). The digital images were displayed by FluorChem Q (Alpha Innotech).

### Mouse PrP-D158 structure modeling

*In silico* PrP mutant N158D models based on the mouse prion protein structures available at the Protein Data Bank archive (www.rcsb.org) [69] were generated using the SWISS-MODEL homology-model [70–74] server at ExPASy. Briefly, a template search with Blast [74] and HHBlits [75] was performed against the SWISS-MODEL template library and models were built using ProMod3 and alternatively PROMOD-II [76]. Following loop and side chains modeling, a last energy minimization step is performed using the OpenMM [77] molecular mechanics library.

For structural analysis purposes two different models were chosen based on different template structures obtained from different experimental methods. Firstly, a NMR-based model (Model01) from mouse prion protein fragment 121–231 at 37°C, PDB ID: 2L39 [78] and a second one based on X-ray crystallography (Model02) from mouse prion protein structure complexed with Promazine PDB ID: 4MA7 [79]. Both models were those reporting best Global Model Quality Estimation (GMQE) [80].

Figures and molecular surfaces with electrostatic potential were produced with PyMol [81].

### Generation of TgN158D mice

The genetic construct containing the mouse N158D substitution was carried out by PCR site-directed mutagenesis which first uses internal primers for the specific substitution: 5’ CATGTACCGCTACCCTGACCAAGTGTACTACAGGCC 3’ and 5’ GGCCTGTAGTACACTTGGTCAGGGTAGCGGTACATG 3’. After isolation by PCR amplification using 5’ CCGGAATTCCGGCGTACGATGGCGAACCTTGGCTAC 3’ and 5’ CTAGTCTAGACTAGGCCGGCCTCATCCCACGATCAGGAAG 3’ as primers, the mouse N158D-PrP ORF was cloned into the pGEM-T vector (Promega) and excised from the cloning vector by using restriction enzymes *BsiWI* (Thermo Fisher Scientific Inc.) and *FseI* (New England Biolabs Ltd.), and then inserted into a modified version of MoPrP.Xho vector [82], as described previously [83], which was also digested with *BstWI* and *FseI*. This vector contains the murine PrP promoter and exon-1, intron-1, exon-2 and 3’ untranslated sequences. The transgene was excised using *NotI* and purified with Invisorb Spin DNA Extraction Kit (Inviteck) according to the manufacturer’s recommendations.

Transgenic mouse founders were generated by microinjection of DNA into pronuclei following standard procedures [83]. DNA extracted from tail biopsies was analyzed by PCR using specific primers for the mouse exon 2 and 3’ untranslated sequences (5’ GAACTGAACCATTTCAACCGAG 3’ and 5’ AGAGCTACAGGTGGATAACC 3’). Those which tested positive were bred to mice null for the mouse *PRNP* gene in order to avoid endogenous expression of mouse prion protein. Absence of the mouse endogenous *PRNP* was assessed using the following primers: 5’ ATGGCGAACCTTGGCTACTGGC 3’ and 5’ GATTATGGGTACCCCCTCCTTGG 3’. The mouse N158D PrP expression levels of brain homogenates from transgenic mouse founders were determined by Western blot using anti-PrP MAb Saf-83 (1:400) and compared with the PrP expression levels from wild-type mouse brain homogenates.

### Cell/brain-PMCA procedures for propagation/inhibition studies

To evaluate the effect of *in vitro* propagation of the residue 158 in mouse PrP, a standard 24 h PMCA, based on mouse brain homogenate mixed with cellular 3F4-tagged PrP as substrate, was performed using 1:40 dilution of RML or 22L as seeds. Four different cell lines expressing different types of 3F4-tagged PrPs were used: mouse N158, D158, E158 or without PrP (Knock-out PrP cells). All of them were obtained by transient expression of the following plasmids on a PrP knock-out cell line (*PRNP*^0/0^ mouse cell line), specifically, the *PRNP*^*0/0*^ hippocampus-derived HpL3-4 cell line (except for the cells devoid of PrP, which were not transfected) [84]. Briefly, mouse genomic DNA was extracted from the brain of a C57BL6 mouse using NucleoSpin tissue kit (Macherey-Nagel) and following the instructions. The ORF from wild-type mouse PrP was obtained by PCR using primers 5’ CCGGAATTCCGGCGTACGATGGCGAACCTTGGCTAC 3’ and 5’ CTAGTCTAGACTAGGCCGGCCTCATCCCACGATCAGGAAG 3’. The PCR product was cloned into a pCMV vector (Thermo Fisher Scientific Inc.) by using *EcoRI* and *NotI* (Thermo Fisher Scientific Inc.) restriction enzymes. Site-directed mutagenesis was used to introduce the 3F4 tag in wild-type mouse PrP using internal primers 5’ CAAACCAAAAACCAACATGAAGCATATGGCAGGGGCTGCGGC 3’ and 5’ GCCGCAGCCCCTGCCATATGCTTCATGTTGGTTTTTGGTTTG 3’. N158D and N158E mutations were also introduced by site-directed mutagenesis using internal primers 5’ GAAAACATGTACCGCTACCCTG 3’ and 5’ CTGGCCTGTAGTACACTTGGTC 3’ and 5’ GAAAACATGTACCGCTACCCTGAG 3’ and 5’ CTGGCCTGTAGTACACTTGCTC 3’, respectively. For all the mutagenesis, the same external primers than those used for wild-type mouse PrP were used and all mutated PrP were cloned into pCMV by using *EcoRI* and *NotI* restriction enzymes. Non-PMCA amplified samples and samples subjected to one 24 h single round of PMCA were digested with 20 μg/ml of PK and analyzed by Western blot using monoclonal antibody 3F4 (1:10,000).

To evaluate the inhibitory effect of the residue 158 in mouse PrP during the *in vitro* propagation of a wild-type mouse PrP, a standard 24 h PMCA, based on mouse brain homogenate mixed with cellular PrP as substrate, was performed using 1:5,000 and 1:10,000 dilutions of RML as seed. Four different cell lines expressing different type of mutated PrPs were used: mouse N158, D158, E158 or without PrP (Knock-out PrP cells). Non-PMCA amplified samples and samples subjected to one 24 h single round of PMCA were digested with 20 μg/ml of PK and analyzed by Western blot using monoclonal antibody D18 (1:10,000).

### Wild-type and TgN158D mice inoculation

C57BL6 and TgN158D mice of 42–56 days of age were inoculated intracerebrally under gaseous anesthesia (Isoflurane) through the right parietal bone. A 50 μl SGC precision syringe was used with a 25 G gauge needle and coupled to a repeatability adaptor fixed at 20 μl. Buprenorphine (0.3 mg/kg) was injected subcutaneously before recovery to consciousness to reduce post-inoculation pain.

Mice were kept in a controlled environment at 22°C, 12 h light -darkness cycle and 60% relative humidity in HEPA filtered cages (both air inflow and extraction) in ventilated racks. The mice were fed *ad libitum*, observed daily and their clinical status assessed twice a week until neurological clinical signs appeared, after which they were examined daily. The presence of ten different TSE-associated clinical signs [82] was scored. Diseased animals were culled at the terminal stage of the disease by exposure to a rising concentration of carbon dioxide. Survival time was calculated as the interval between inoculation and culling or death. Brains were removed immediately after death, if euthanized/culled, and divided into two parts; a portion was stored at -80°C and the other fixed in 10% formalin for histological studies.

### Neuropathology

Fixed tissues were dehydrated through increasing alcohol concentrations, through xylene and then embedded in paraffin-wax. Four micrometer sections were mounted on glass microscope slides and stained with hematoxylin-eosin for morphological evaluation. Further slides were mounted on 3-trietoxysilil-propilamine-coated glass slides for immunohistochemical studies.

Immunohistochemistry (IHC) for disease associated PrP was performed. Briefly, deparaffinised sections were immersed in formic acid and boiled at low pH in a pressure cooker, endogenous peroxidases were blocked, the sections were pre-treated with Protease-K (PK) and incubated overnight with the primary antibody (anti-PrP mAb 6H4, Prionics AG, 1:1000 diluted in DAKO background reducing antibody diluent). Finally, the DAKO EnVision system was used along with 3,3’diaminobenzidine (as the chromogen substrate) to visualize any PrP deposits. For cellular PrP staining, the formic acid step was omitted and a standard heat induced epitope retrieval step (20 min at 95°C) was performed using DAKO Target Retrieval Solution. The same antibody was used but at a 1:100 dilution.

### Ethics statement

All experiments involving animals adhered to the guidelines included in the Spanish Legislative Decree “Real Decreto 1201/2005 de 10 de Octubre” on protection of animals used for experimentation and other scientific purposes, which transposed the European Directive 86/609/EEC on Laboratory Animal Protection. The project was approved by the Ethical Committee on Animal Welfare of the Laboratorio Central de Veterinaria (project code assigned by the Ethical Committee CEBA-07/2010) was performed under its supervision.

## Supporting information

S1 Fig Level of PrP^C^ expression in brains from different species. To compare the PrP^C^ expression levels present in the brains from different species a serial dilution (1:4, 1:8, 1:16 and 1:32 and 1:64) was performed. *Dog 1*: Cocker Spaniel. *Dog 2*: German Wirehaired Pointer. *Rabbit*: New Zealand white rabbit. *Cat*: European shorthaired. *Mouse*: C57BL6 mouse. Undigested samples were analyzed by Western blot using monoclonal antibody POM1 (1:5,000). Equivalent amount of PrP was observed in all the samples. Normal brain homogenates were run in separate gels and the image shown, divided by vertical grey lines. Mw: Molecular weight.
(TIF)

S2 Fig PrP protein amino acid alignment (residues 91–230, dog PrP numbering) from several dog breeds. *: Heterozygous Ser-Gly at position 101. Identical amino acids are indicated by dots. Accession numbers: doPrP (FJ870767.1); Cocker Spaniel (KY649551); German Wirehaired Pointer (KY649553); Australian Shepherd (KY649552); Pyrenean Shepherd (KY649554); Belgian Malinois Shepherd (KY649555); White Shepherd (KY649556); Schipperke (KY649557); Belgian Tervuren Shepherd (KY649558); Old English Sheepdog (KY649559); Border Collie (KY649560); Pointer (KY649561); Standard Poodle (KY649562); Doberman Pinscher (KY649563). Amino acid numbers refer to dog PrP (doPrP).
(TIF)

S3 Fig PrP sequence alignments. **A**. PrP protein amino acid (91–230) alignment (dog PrP numbering) of GenBank published sequences containing Asp or Glu at codon 163. The residues present at positions 101 and 163 are highlighted. Two different sequences for coyote differing at codon 101, and 4 different sequences for dog differing at codons 101 and 163 are shown. Wolverine and pine marten differ in 6 amino acid residues with canid PrP but not at codon 163. Identical amino acids are indicated by dots. **B**. Phylogenetic tree based on the PrP 91–230 sequences shown in A. Line distances are arbitrary and not relative to divergence times. The presence of Asp at codon 163 might be explained for canids and some mustelids as a consequence of a shared ancestor. Species codification and accession numbers: doPrP S_101_/D_163_, dog PrP (FJ870767); gwPrP S_101_/D_163_, gray wolf PrP (AGA63688); bdPrP S_101_/D_163_, bush dog (AGA63670); coPrP S_101_/D_163_, coyote (AGA63673); mwPrP S_101_/D_163_, maned wolf PrP (AGA63698); doPrP G_101_/D_163_, dog PrP (AGA63678); rdPrP G_101_/D_163_, raccoon dog (ACA50735); coPrP G_101_/D_163_, coyote (ACJ06781); sfPrP G_101_/D_163_, swift fox (ACA50741); rfPrP G_101_/D_163_, red fox (AGA63703); afPrP G_101_/D_163_, artic fox (ABY66540); diPrP G_101_/D_163_, dingo PrP (AAD12061); doPrP G_101_/E_163_, dog PrP (ABL75506); doPrP S_101_/E_163_, dog PrP (NP_001013441.1); wvPrP G_101_/D_163_, woverine PrP (AGA63709); maPrP G_101_/D_163_, pine marten PrP (AGA63684). **C**. Phylogenetic tree based on taxonomic opinion, phenotypic data and primarily DNA sequence data. Adapted from Nyakatura and Bininda-Emonds, 2012 [85]. Line distances are arbitrary and not relative to divergence times. Magnification of the evolutionary relationships among canids and mustelids is shown.
(TIF)

S4 Fig Local environments of mouse N158 from PrP PDB ID 1XYX (gray); PDB ID 2L39 (dark pink) & PDB ID 4MA8 (light pink) and D158 (equivalent to position 163 from canine PrP) PDB ID 1XYK (green) structures and conformers for Arg135 (R135) in the same datasets. Residue numbers for R135 are labeled with the PDB code as sticks. Comparison of local environments at N158/D158, suggests that the R135-D158 interaction depends mainly on the R135 side chain rotamer as observed in wild-type mouse prion structures.
(TIF)

S5 Fig PrP expression levels of Tg402 and Tg403 compared to C57BL/6 and Tga20 mice. 10% brain homogenates from C57BL/6, Tg402 and Tg403 mice were diluted 1:20, 1:40, 1:80, 1:160 and 1:320 and diluted 10% brain homogenate from Tga20 diluted 1:40, 1:80, 1:160, 1:320 and 1:640 were analyzed by Western blot using monoclonal antibody Saf-83 (1:400). The PrP expression levels of Tg402 and Tg403 are approximately 1x and 2x, respectively, compared to C57BL/6. PrP expression levels of Tga20 are about 8 to 10x higher than C57BL/6.
(TIF)

S6 Fig Remnant signal of BSE-C inoculum not submitted to PMCA and demonstration that cattle PrP^C^ present in the inocula does not sustain BSE-C propagation. Western blot showing the same 1:3 dilution of the BSE-C (isolate 1) seed used in dog brain homogenates submitted to PMCA (procedure D1). The lack of remnant signal of the seed applied at 30% on the substrate (final 1:3 dilution) and the new signal appearing just when cow but not *Prnp*^*0/0*^ brain homogenate (KO) was used, suggests: i. the BSE-C (isolate 1) used as seed for *in vitro* propagations shows a low PrP^res^ signal (after Protease-K treatment) that almost disappears after a 1:3 dilution, and ii. the remaining cattle PrP^C^ present in the seed does not sustain BSE-C propagation when a substrate based on *Prnp*^*0/0*^ brain homogenate (KO) is used. Samples were treated with 85 μg/ml of Protease-K (PK) and protease resistant proteins were analyzed by Western blot using D18 (1:2,000) monoclonal antibody. Cont.: Undigested cow brain homogenate.
(TIF)

S7 Fig PrP expression levels of transiently transfected cells compared to PrP expression levels in C57BL/6 mouse brain homogenate. 10% C57BL/6 mouse brain homogenate was diluted (1:1, 1:2, 1:4, 1:8, 1:16 and 1:32) equal to the homogenates from cells transfected with the following plasmids: pCMV N158 (wt) mouse PrP, pCMV E158 mouse PrP and pCMV D158 mouse PrP. Cell homogenates mixed with the brain homogenate were used as substrates for cell PMCA experiments. All the samples were analyzed with monoclonal antibody D18 (1:10,000). PrP^C^ levels in cell homogenates are approximately three times lower than the levels from the mouse brain homogenate. PK: Protease-K. Mw: Molecular weight.
(TIF)

S8 Fig Immunohistochemical analysis of PrP^C^ expression in homozygous Tg402 and Tg403 compared to C57BL/6. Cerebral cortex sections from Tg402, Tg403 and C57BL/6 mice were used to compare the localization of PrP^C^ expression. A fine granular neuropil immunolabeling (corresponding to PrP^C^ on the dendrite cell membrane) and absence of labeling within the pericarion were observed. PrP^C^ immunolabeling from homozygous Tg402 and Tg403 brains was comparable to that found in WT (C57BL/6) brains but more intense, due to a slight overexpression. Samples were immunostained using 6H4 (1:100) monoclonal antibody. Bar: 25 μm.
(TIF)
