# Supplementary figures and images for: Unraveling the key to the resistance of canids to prion diseases
Source: PLoS Pathog. 2017 Nov 13;13(11):e1006716. doi: 10.1371/journal.ppat.1006716 (PMC5703577; doi:10.1371/journal.ppat.1006716)

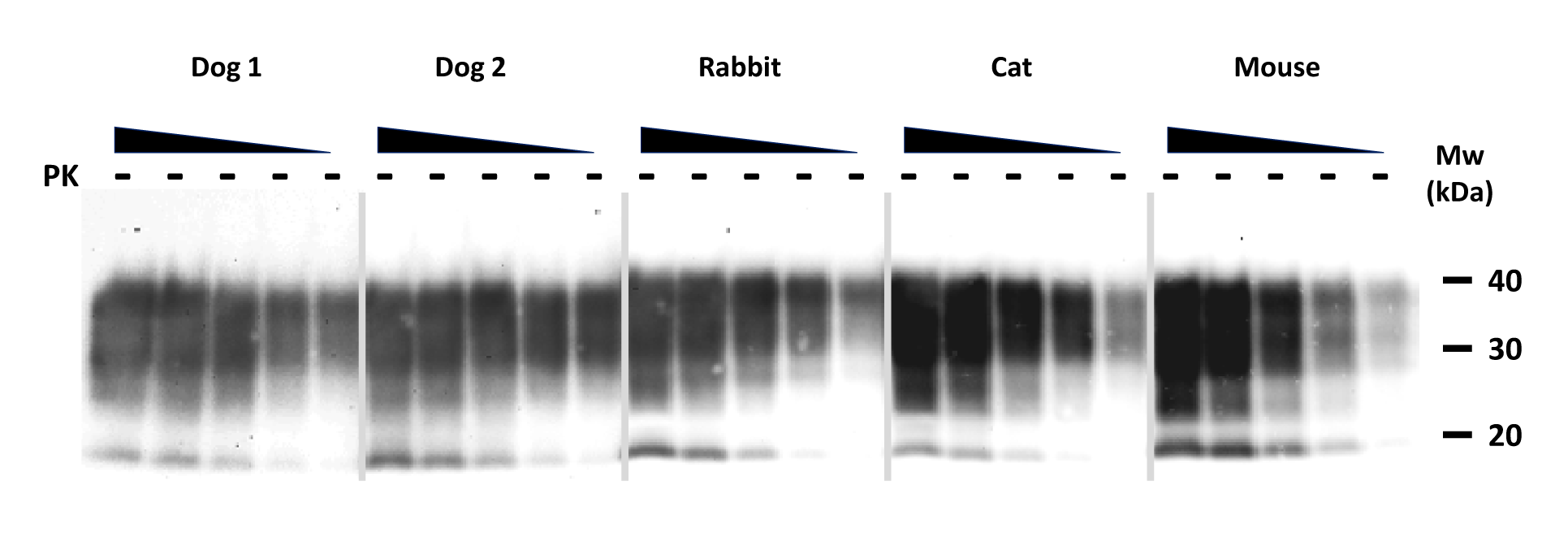

Supplement: S1 Fig — To compare the PrPC expression levels present in the brains from different species a serial dilution (1:4, 1:8, 1:16 and 1:32 and 1:64) was performed. Dog 1: Cocker Spaniel. Dog 2: German Wirehaired Pointer. Rabbit: New Zealand white rabbit. Cat: European shorthaired. Mouse: C57BL6 mouse. Undigested samples were analyzed by Western blot using monoclonal antibody POM1 (1:5,000). Equivalent amount of PrP was observed in all the samples. Normal brain homogenates were run in separate gels and the image shown, divided by vertical grey lines. Mw: Molecular weight. (TIF) [file ppat.1006716.s001.tif]

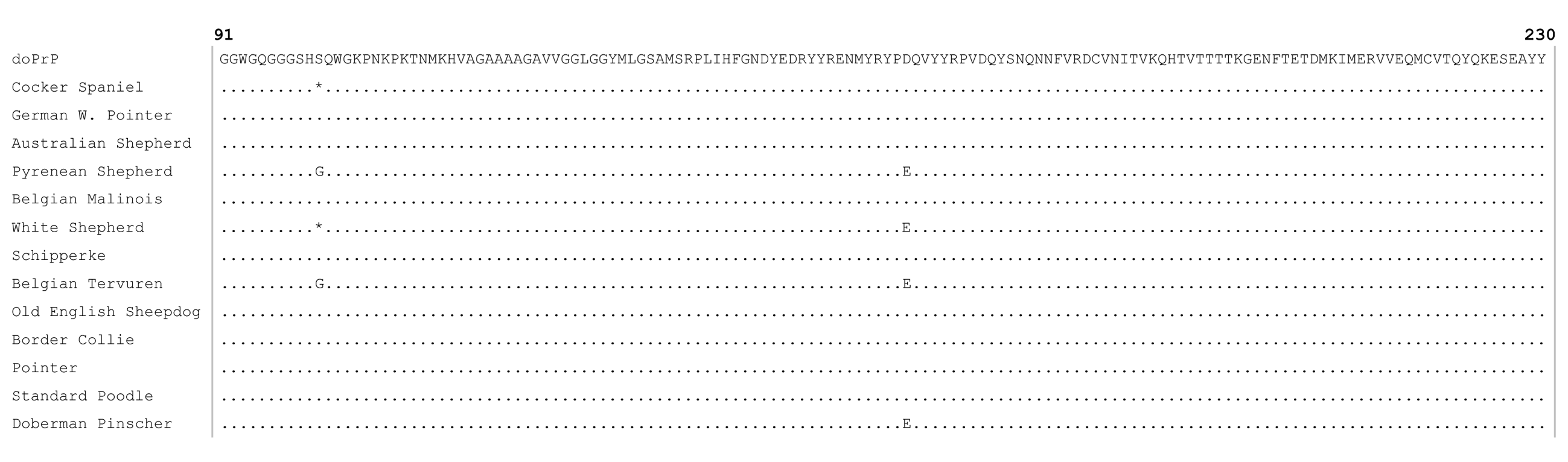

Supplement: S2 Fig — *: Heterozygous Ser-Gly at position 101. Identical amino acids are indicated by dots. Accession numbers: doPrP (FJ870767.1); Cocker Spaniel (KY649551); German Wirehaired Pointer (KY649553); Australian Shepherd (KY649552); Pyrenean Shepherd (KY649554); Belgian Malinois Shepherd (KY649555); White Shepherd (KY649556); Schipperke (KY649557); Belgian Tervuren Shepherd (KY649558); Old English Sheepdog (KY649559); Border Collie (KY649560); Pointer (KY649561); Standard Poodle (KY649562); Doberman Pinscher (KY649563). Amino acid numbers refer to dog PrP (doPrP). (TIF) [file ppat.1006716.s002.tif]

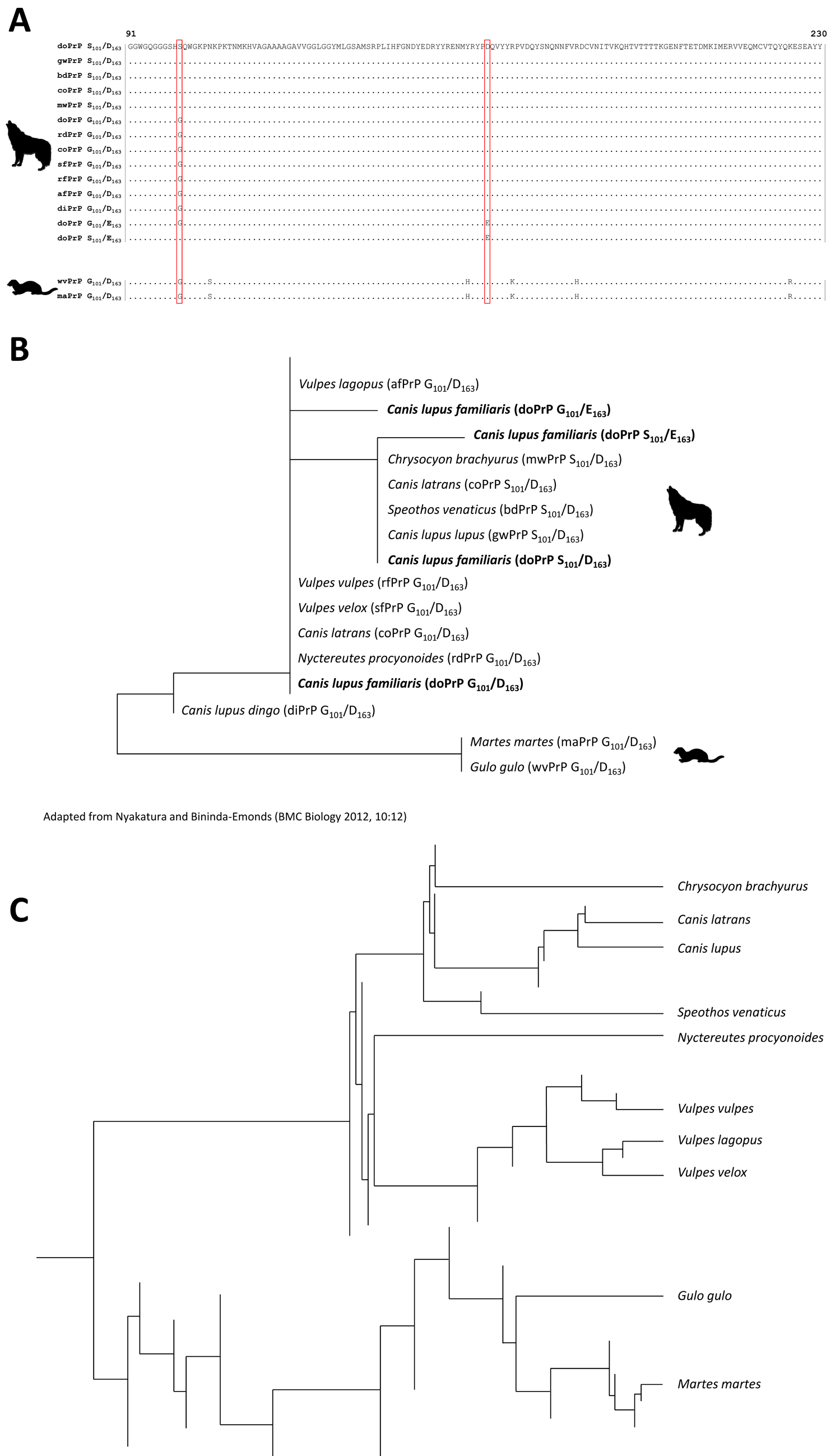

Supplement: S3 Fig — A. PrP protein amino acid (91–230) alignment (dog PrP numbering) of GenBank published sequences containing Asp or Glu at codon 163. The residues present at positions 101 and 163 are highlighted. Two different sequences for coyote differing at codon 101, and 4 different sequences for dog differing at codons 101 and 163 are shown. Wolverine and pine marten differ in 6 amino acid residues with canid PrP but not at codon 163. Identical amino acids are indicated by dots. B. Phylogenetic tree based on the PrP 91–230 sequences shown in A. Line distances are arbitrary and not relative to divergence times. The presence of Asp at codon 163 might be explained for canids and some mustelids as a consequence of a shared ancestor. Species codification and accession numbers: doPrP S101/D163, dog PrP (FJ870767); gwPrP S101/D163, gray wolf PrP (AGA63688); bdPrP S101/D163, bush dog (AGA63670); coPrP S101/D163, coyote (AGA63673); mwPrP S101/D163, maned wolf PrP (AGA63698); doPrP G101/D163, dog PrP (AGA63678); rdPrP G101/D163, raccoon dog (ACA50735); coPrP G101/D163, coyote (ACJ06781); sfPrP G101/D163, swift fox (ACA50741); rfPrP G101/D163, red fox (AGA63703); afPrP G101/D163, artic fox (ABY66540); diPrP G101/D163, dingo PrP (AAD12061); doPrP G101/E163, dog PrP (ABL75506); doPrP S101/E163, dog PrP (NP_001013441.1); wvPrP G101/D163, woverine PrP (AGA63709); maPrP G101/D163, pine marten PrP (AGA63684). C. Phylogenetic tree based on taxonomic opinion, phenotypic data and primarily DNA sequence data. Adapted from Nyakatura and Bininda-Emonds, 2012 [85]. Line distances are arbitrary and not relative to divergence times. Magnification of the evolutionary relationships among canids and mustelids is shown. (TIF) [file ppat.1006716.s003.tif]

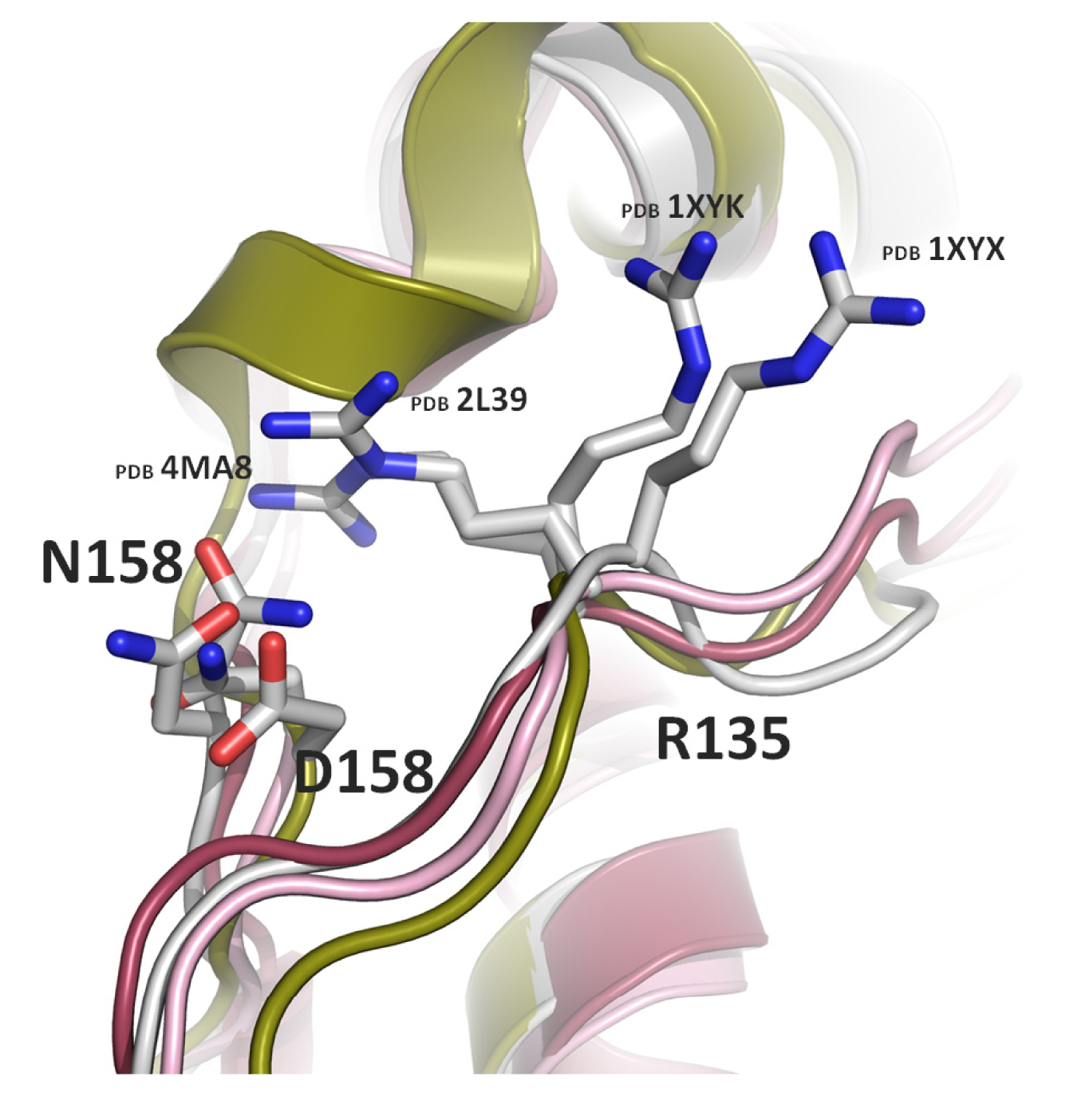

Supplement: S4 Fig — Residue numbers for R135 are labeled with the PDB code as sticks. Comparison of local environments at N158/D158, suggests that the R135-D158 interaction depends mainly on the R135 side chain rotamer as observed in wild-type mouse prion structures. (TIF) [file ppat.1006716.s004.tif]

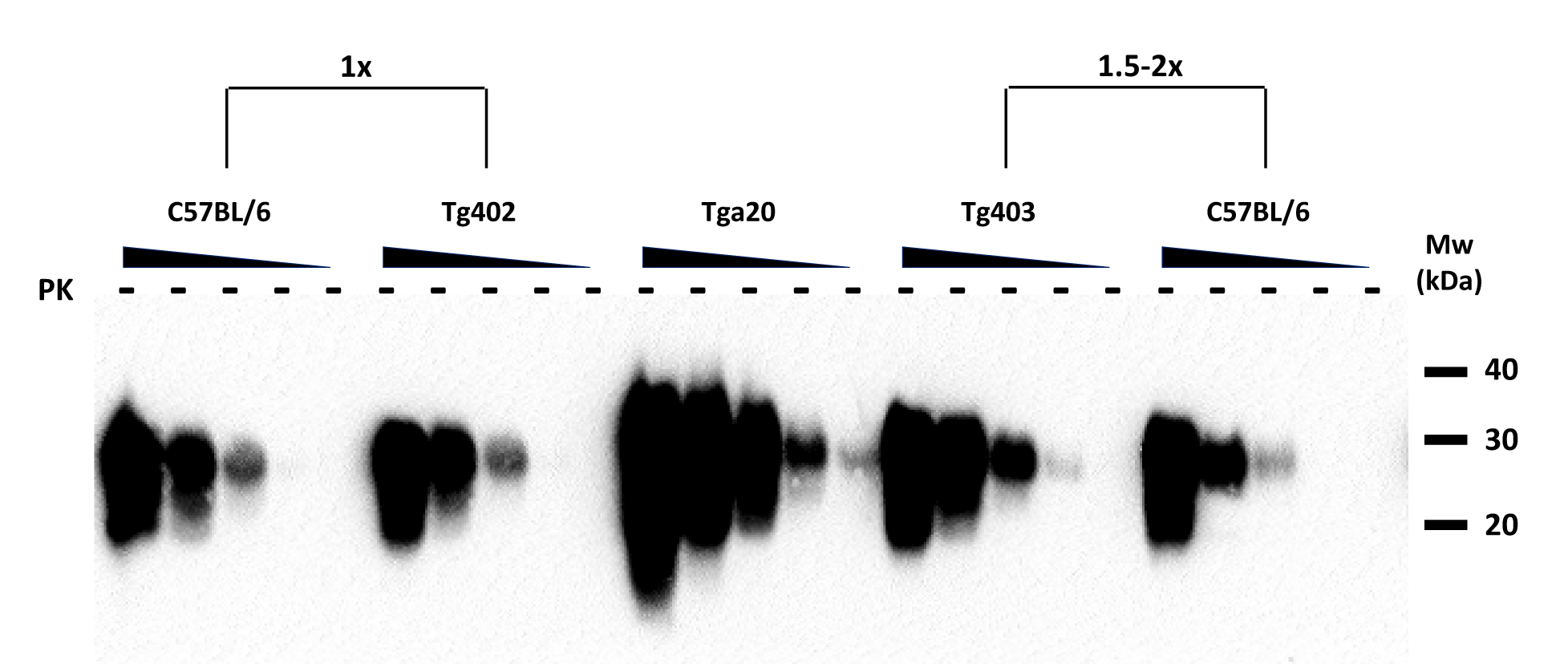

Supplement: S5 Fig — 10% brain homogenates from C57BL/6, Tg402 and Tg403 mice were diluted 1:20, 1:40, 1:80, 1:160 and 1:320 and diluted 10% brain homogenate from Tga20 diluted 1:40, 1:80, 1:160, 1:320 and 1:640 were analyzed by Western blot using monoclonal antibody Saf-83 (1:400). The PrP expression levels of Tg402 and Tg403 are approximately 1x and 2x, respectively, compared to C57BL/6. PrP expression levels of Tga20 are about 8 to 10x higher than C57BL/6. (TIF) [file ppat.1006716.s005.tif]

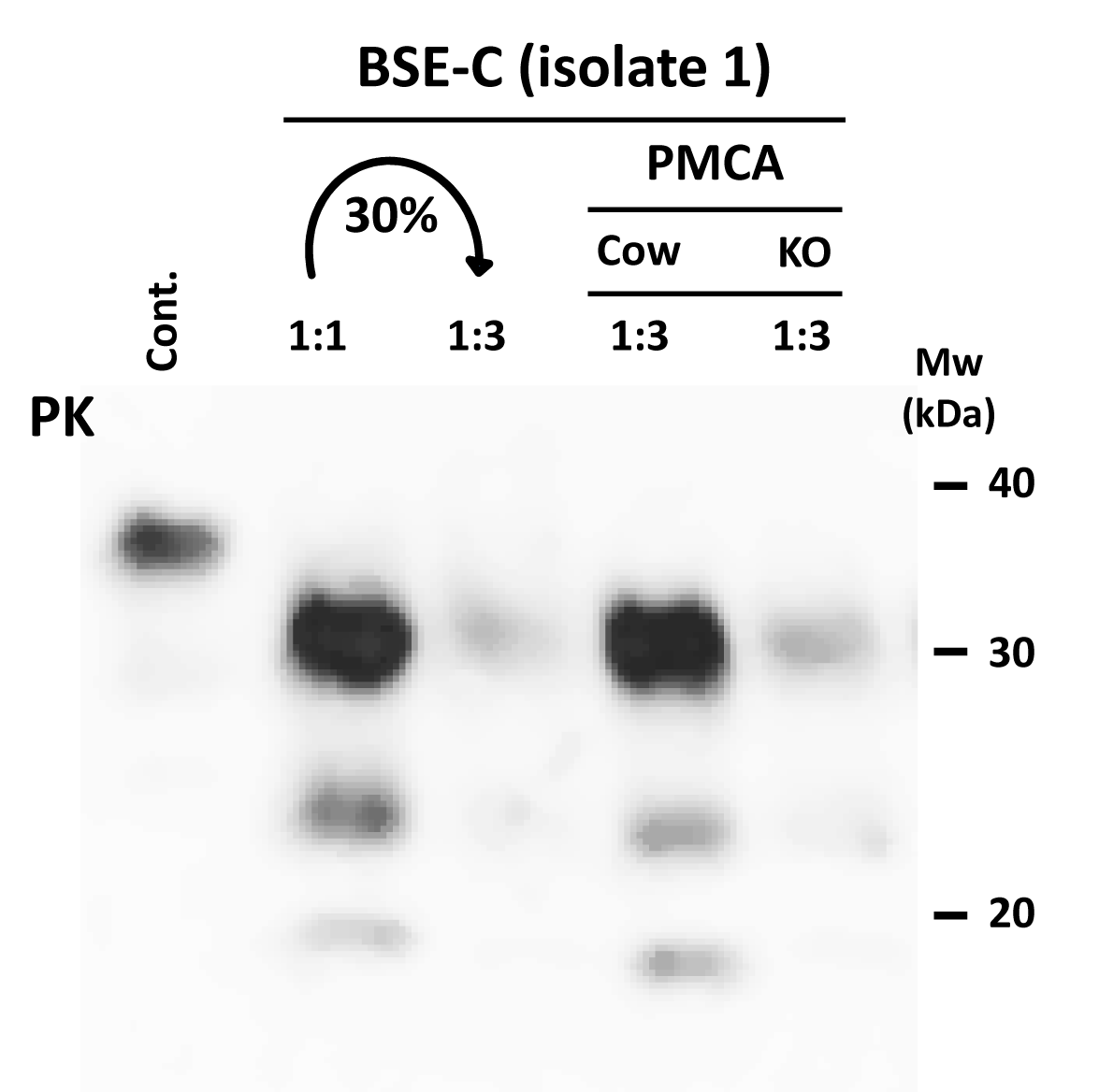

Supplement: S6 Fig — Western blot showing the same 1:3 dilution of the BSE-C (isolate 1) seed used in dog brain homogenates submitted to PMCA (procedure D1). The lack of remnant signal of the seed applied at 30% on the substrate (final 1:3 dilution) and the new signal appearing just when cow but not Prnp0/0 brain homogenate (KO) was used, suggests: i. the BSE-C (isolate 1) used as seed for in vitro propagations shows a low PrPres signal (after Protease-K treatment) that almost disappears after a 1:3 dilution, and ii. the remaining cattle PrPC present in the seed does not sustain BSE-C propagation when a substrate based on Prnp0/0 brain homogenate (KO) is used. Samples were treated with 85 μg/ml of Protease-K (PK) and protease resistant proteins were analyzed by Western blot using D18 (1:2,000) monoclonal antibody. Cont.: Undigested cow brain homogenate. (TIF) [file ppat.1006716.s006.tif]

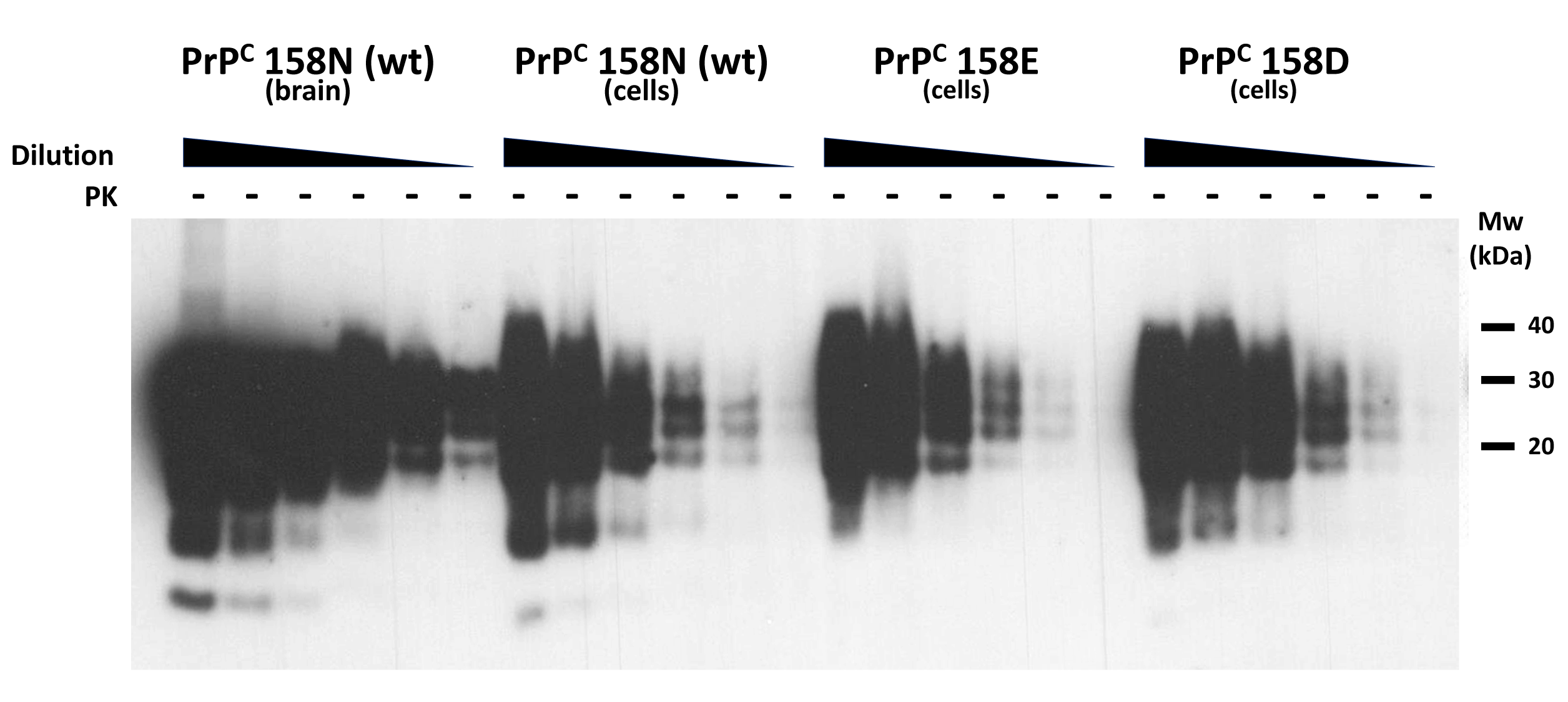

Supplement: S7 Fig — 10% C57BL/6 mouse brain homogenate was diluted (1:1, 1:2, 1:4, 1:8, 1:16 and 1:32) equal to the homogenates from cells transfected with the following plasmids: pCMV N158 (wt) mouse PrP, pCMV E158 mouse PrP and pCMV D158 mouse PrP. Cell homogenates mixed with the brain homogenate were used as substrates for cell PMCA experiments. All the samples were analyzed with monoclonal antibody D18 (1:10,000). PrPC levels in cell homogenates are approximately three times lower than the levels from the mouse brain homogenate. PK: Protease-K. Mw: Molecular weight. (TIF) [file ppat.1006716.s007.tif]

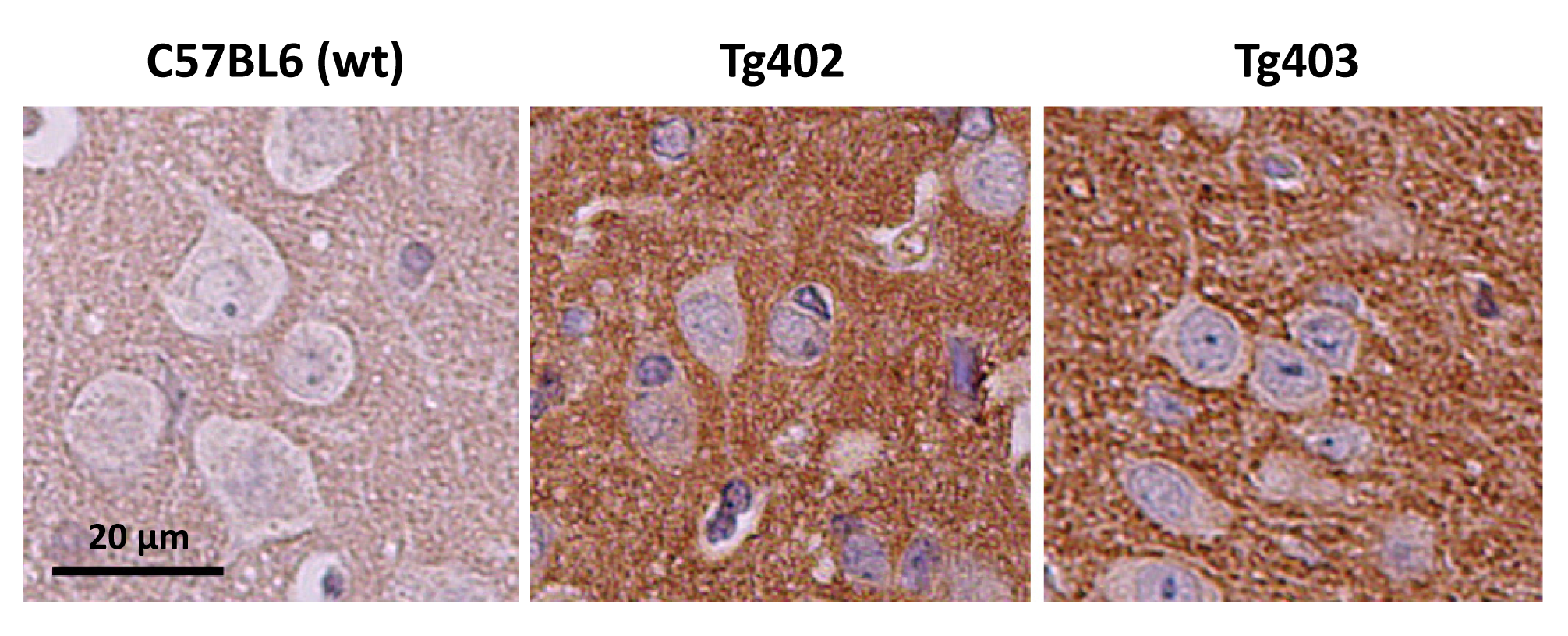

Supplement: S8 Fig — Cerebral cortex sections from Tg402, Tg403 and C57BL/6 mice were used to compare the localization of PrPC expression. A fine granular neuropil immunolabeling (corresponding to PrPC on the dendrite cell membrane) and absence of labeling within the pericarion were observed. PrPC immunolabeling from homozygous Tg402 and Tg403 brains was comparable to that found in WT (C57BL/6) brains but more intense, due to a slight overexpression. Samples were immunostained using 6H4 (1:100) monoclonal antibody. Bar: 25 μm. (TIF) [file ppat.1006716.s008.tif]
